# Whole genome sequencing reveals environmental pathogen misidentification and potential for cross-phylum antimicrobial resistance gene transfer in bovine mastitis: a pilot genomic study

**DOI:** 10.1186/s12917-025-05280-z

**Published:** 2026-01-14

**Authors:** A. Muhee, A. Pandit, Sobby Jan, Iqra Shafi Khan, Nuzhat Hassan, R. A. Bhat, M. I. Yatoo

**Affiliations:** 1 Division of Veterinary Clinical Complex, FVSc & AH, Shuhama, Sher E Kashmir University of Agricultural Sciences and Technology of Kashmir, Srinagar, Jammu and Kashmir India; 2 Mountain Livestock Research Institute, Sher E Kashmir University of Agricultural Sciences and Technology of Kashmir, Srinagar, Jammu and Kashmir India; 3 Division of Veterinary Epidemiology and Preventive Medicine, FVSc & AH, Shuhama, Sher E Kashmir University of Agricultural Sciences and Technology of Kashmir, Srinagar, Jammu and Kashmir India

**Keywords:** Bovine mastitis, Whole genome sequencing, Antimicrobial resistance, Stutzerimonas stutzeri, Diagnostic limitations, Horizontal gene transfer, Dairy cattle, Veterinary microbiology

## Abstract

**Background:**

The diagnosis of bovine mastitis relies predominantly on conventional microbiological methods optimized for common pathogens, potentially ignoring environmental bacteria with complex antimicrobial resistance profiles.

**Methods:**

This pilot study combined conventional identification with whole genome sequencing (WGS) analysis of bovine mastitis isolates. A total of 330 milk samples were analyzed using standard microbiological methods, followed by comprehensive genomic characterization of two representative multidrug-resistant isolates using Illumina NovaSeq 6000 sequencing. The limited sample size reflects the pilot nature of this proof-of-concept study. Analysis of antimicrobial resistance genes was performed using BLAST searches against the Comprehensive Antibiotic Resistance Database.

**Results:**

Of 330 samples, 202 (61.2%) tested positive for mastitis. WGS revealed misidentification of critical species of an environmental pathogen (Stutzerimonas stutzeri) and comparative analysis with E. coli (included as a control for a known mastitis pathogen). An isolate originally characterized as Gram-positive with Staphylococcus-like morphology was definitively identified as Stutzerimonas stutzeri by genomic analysis. Both isolates harbored diverse antimicrobial resistance genes with phylogenetic origins spanning multiple bacterial orders and phyla (Enterobacterales, Bacillales, Pseudomonadales, Enterococcales), suggesting a potential for horizontal gene transfer. Mobile genetic elements such as plasmids, integrons and insertion sequences were identified in both genomes, consistent with the ability for gene mobility. Phylogenetic analysis revealed that resistance genes originated from Proteobacteria (61%) and Firmicutes (39%), indicating cross-phylum gene exchange.

**Conclusions:**

This pilot study provides preliminary evidence that whole genome sequencing can identify bacterial species that may be missed by traditional diagnostic methods. Analysis of two isolates revealed evidence of horizontal gene transfer potential in mastitis-associated bacteria. The environmental pathogen S. stutzeri may represent a poorly recognized opportunistic mastitis pathogen with significant resistance potential. Based on these exploratory findings from two cases, our results suggest the potential utility of genomic surveillance approaches in veterinary diagnostic microbiology, necessitating larger validation studies.

**Supplementary Information:**

The online version contains supplementary material available at 10.1186/s12917-025-05280-z.

## Highlights

i. WGS identified misclassified environmental pathogen in bovine mastitis.

ii. S. stutzeri showed extensive AMR genes despite conventional ID failure.

iii. Phylogenetic diversity and mobile elements suggest horizontal gene transfer potential.

iv. Genomic analysis revealed 287-294 resistance genes per isolate.

v. Pilot study demonstrates need for molecular diagnostic approaches.

**Supplementary Information:**

The online version contains supplementary material available at 10.1186/s12917-025-05280-z.

## Background

 Current veterinary diagnostic paradigms for bovine mastitis rely heavily on morphological and biochemical identification methods developed for common pathogens, creating systematic bias toward expected organisms while potentially missing environmental bacteria with clinical significance [20, 30]. The advent of whole-genome sequencing (WGS) offers unbiased species identification capabilities that can reveal the true microbial diversity in clinical infections, particularly for organisms that may exhibit atypical phenotypic characteristics in host environments.

Bovine mastitis remains the most economically significant disease affecting dairy cattle worldwide, causing substantial losses through reduced milk production, increased treatment costs, and premature culling [29]. However, the full spectrum of causative organisms may be underestimated due to limitations in conventional identification approaches [18]. Traditional identification approaches, which rely on morphological characteristics, biochemical tests, and targeted PCR amplification, are optimized for common mastitis pathogens, such as *Staphylococcus aureus*, *Escherichia coli*, and *Streptococcus* species, potentially overlooking emerging or atypical bacterial species [30].

The limitations of conventional identification methods are particularly evident when investigating complex microbial communities in mastitis-affected milk. Studies using culture-independent approaches have revealed significant microbial diversity beyond traditionally recognized mastitis pathogens, including environmental bacteria that may contribute to infection dynamics and antimicrobial resistance dissemination [8, 10]. This hidden diversity has important implications for understanding the evolution of resistance, as environmental bacteria often serve as reservoirs for antimicrobial resistance genes that can be transferred to clinical pathogens through horizontal gene transfer mechanisms [9].

Whole-genome sequencing (WGS) has emerged as a transformative tool that addresses the limitations of conventional identification and provides comprehensive insights into antimicrobial resistance mechanisms, virulence factors, and evolutionary relationships [17]. Unlike targeted approaches, WGS enables unbiased species identification and can reveal the complete genomic context of resistance determinants, including mobile genetic elements, that facilitate the spread of resistance [7]. This technology has proven particularly valuable in veterinary microbiology, where accurate pathogen identification directly affects treatment decisions and resistance surveillance programs [12].

Environmental bacteria that cause mastitis may exhibit phenotypic plasticity in host-associated environments, leading to misclassification when relying solely on conventional diagnostic methods. This diagnostic challenge is particularly relevant in regions with limited access to advanced molecular diagnostic tools, where treatment decisions depend heavily on accurate pathogen identification. The widespread use of antimicrobials in dairy farming raises concerns about the evolution and spread of resistance, particularly given the potential for resistance gene transfer between diverse bacterial species sharing the same ecological niche (Jl et al., 2015) [16].

This pilot study addresses these methodological limitations by combining conventional microbiological surveillance with whole-genome sequencing analysis to evaluate diagnostic accuracy and characterize the genomic diversity of mastitis-associated bacteria.

Our main objective was to examine cases in which standard diagnostic approaches produced ambiguous results. First, we aim to determine the prevalence and distribution of mastitis pathogens using traditional identification methods, (2) perform comprehensive genomic characterization of selected isolates using whole genome sequencing (WGS), (3) compare traditional identification results with WGS-based species identification, (4) characterize antimicrobial resistance (AMR) gene profiles and correlate them with phenotypic resistance patterns, and (5) assess horizontal gene transfer potential by identifying mobile genetic elements evaluate. This integrated approach allows for the assessment of both current surveillance capabilities and the potential benefits of implementing genomic technologies in veterinary diagnostic laboratories.

## Methods

### Study design and sample collection

This cross-sectional pilot study was conducted in Kashmir Valley, Jammu and Kashmir, India, from March 2023 to September 2023. The study protocol was approved by the Institutional Animal Ethics Committee of Sher-e-Kashmir University of Agricultural Sciences and Technology-Kashmir. Written informed consent was obtained from all participating dairy farmers.

### Sampling strategy and geographic distribution

Samples were collected using stratified random sampling from three different sources to ensure geographic representation and to capture both clinic-based and farm-based mastitis cases: (1) clinical mastitis cases presenting for treatment at the Veterinary Clinical Complex, FVSc & AH, Shuhama (*n* = 124); (2) lactating cows maintained at the dairy farm of Mountain Livestock Research Institute, Manasbal (*n* = 88, sampling on two separate visits); and (3) cases from district veterinary hospitals and dispensaries across the Kashmir Valley (*n* = 118), including Anantnag District Veterinary Hospital (*n* = 54), Srinagar Central Veterinary Hospital (*n* = 36) and Gulab Bagh Veterinary Center (*n* = 28). This multi-site approach captured diverse population contexts, including acute clinical presentations, routine agricultural surveillance, and regional veterinary service networks. One sample was taken per lactating cow (*n* = 330 animals in total).

Clinical mastitis was diagnosed based on udder inflammation, altered milk consistency, and a positive California Mastitis Test (CMT). Subclinical mastitis (somatic cell count > 200,000 cells/mL without clinical signs) was identified using CMT, electrical conductivity measurements, pH testing, and white-side tests.

The udder teats were cleaned and disinfected with 70% ethanol. The first milk streams were discarded, and approximately 15 mL of milk was collected in sterile screw-capped tubes. The samples were immediately placed on ice and transported to the laboratory within 4 h for processing.

### Bacterial isolation and identification

Milk samples were cultured on 5% sheep blood agar, MacConkey agar, and mannitol salt agar plates, and incubated aerobically at 37 °C for 24–48 h. Bacterial isolates were initially identified using conventional biochemical tests including Gram staining, catalase, coagulase, and species-specific identification kits (HiStaph™ and HiStrep™ identification systems (HiMedia Laboratories, Mumbai, India) following manufacturer protocols.

For isolates with staphylococcal morphology, molecular confirmation was attempted using species-specific PCR amplification targeting the nuc gene of Staphylococcus aureus. Additional species-specific PCR was performed targeting the 16 S rRNA gene for E. coli identification and specific for Streptococcus dysgalactiae (Table 1). However, some isolates displaying gram-positive morphology and catalase-positive characteristics yielded inconsistent or negative results with species-specific primers despite repeated testing, necessitating further molecular characterization through WGS. This phenotype-genotype mismatch was a primary criterion for WGS selection.

**Table 1:** PCR primers used for species-specific identification of mastitis pathogens

S.No	Primer Name	Primer Sequence 5’ to 3’	No. of Bases
1.	S.aureus (nuc gene F)	GCGATTGATGGTGATACGGTT	21
2.	S.aureus (nuc gene R)	AGCCAAGCCTTGACGAACTAAAGC	24
3.	S.aureus (Mec A MRS1)	AAAATCGATGGTAAAGGTTGGC	22
4.	S.aureus (Mec A MRS2)	AGTTCTGCAGTACCGGATTTGC	22
5.	E.coli 16SrRNA gene (F)	GACCTCGGTTTAGTTCACAGA	21
6.	E.coli16SrRNAgene (R)	CACACGCTGACGCTGACCA	19
7.	S. dysgalactiea STRD-DyI (F)	GAACACGTTAGGGTCGTC	18
8.	S.dysgalactiea STRD-DyII (R)	AGTATATCTTAACTAGAAAAACTATTG	27

From 202 mastitis-positive samples, bacterial isolation was successful in 202 samples, yielding a minimum of 252 bacterial isolates. Pure cultures (single species) were obtained from 152 samples (95 S. aureus, 14 E. coli, and 8 S. dysgalactiae from both clinical and subclinical cases combined), while mixed infections (presence of ≥ 2 distinct bacterial species) were identified in 50 samples (41 clinical, 9 subclinical) based on distinct colony morphologies on differential media. Each mixed infection sample yielded a minimum of 2 isolates. This targeted selection strategy, while resulting in a small sample size (*n* = 2, representing 0.99% of the 202 positive samples), was designed to address specific research objectives of this study. The proof-of-concept nature of this study prioritized depth of genomic characterization over breadth of sampling.

### Antimicrobial susceptibility testing

Phenotypic antimicrobial susceptibility testing was performed using the disk diffusion method, according to the Clinical and Laboratory Standards Institute (CLSI) guidelines. Bacterial suspensions equivalent to the McFarland standard (0.5) were inoculated onto Mueller-Hinton agar plates. The antimicrobial disks tested included penicillin G (10 units), amoxicillin-clavulanic acid (30 µg), gentamicin (30 µg), cefpodoxime (10 µg), tetracycline (30 µg), streptomycin (10 µg), ceftriaxone (30 µg), enrofloxacin (10 µg), and cefotaxime (30 µg). Plates were incubated at 37 °C for 18–24 h, and inhibition zones were measured and interpreted according to the CLSI breakpoints. Multidrug resistance was defined as the resistance to three or more antimicrobials.

### Sample selection for whole genome sequencing

From 202 mastitis-positive samples, two isolates (0.99%) were selected for comprehensive WGS based on predefined criteria designed to identify diagnostically problematic cases: (1) clinical significance from severe mastitis cases with marked local and systemic signs requiring aggressive treatment, (2) phenotypic multidrug resistance to ≥ 3 antimicrobial classes by disk diffusion testing, (3) ambiguous or discrepant conventional identification result particularly one isolate displaying gram-positive morphology and catalase-positive phenotype but yielding negative results with Staphylococcus species-specific PCR primers, suggesting potential misidentification, (4) representation of different ecological origins (environmental opportunist vs. known enteric pathogen), (5) geographic diversity across sampling sites to avoid single-location bias, and (6) high-quality genomic DNA suitable for Illumina sequencing.

This represents a pilot genomic characterization study (0.99% of positive samples) designed to establish proof-of-concept for WGS-based identification of diagnostic discrepancies rather than comprehensive pathogen surveillance. The limited sample size reflects resource constraints typical of pilot studies and strategic focus on isolates with ambiguous conventional identification results. While this approach does not capture the full diversity of mastitis pathogens, it enables in-depth genomic characterization to validate the utility of WGS for identifying systematically misclassified organisms. The findings should be interpreted within this limited scope, and larger-scale studies are warranted to determine the prevalence of misidentification across broader pathogen populations.

### DNA extraction and quality assessment

Genomic DNA was extracted using the DNeasy Blood & Tissue Kit (Qiagen, Germany) following the manufacturer’s protocol, with modifications for gram-positive bacteria. DNA concentration was quantified using a Qubit 4.0 Fluorometer (Thermo Fisher Scientific), and quality was assessed using 1.0% agarose gel electrophoresis and NanoDrop spectrophotometry. DNA samples with A260/A280 ratios > 1.8 and concentrations > 50 ng/µL were considered suitable for library preparation.

### Library preparation and whole genome sequencing

Paired-end sequencing libraries were prepared using the Twist NGS Library Preparation Kit for Illumina following the manufacturer’s protocol. The workflow includes enzymatic DNA fragmentation, end repair, A-tailing, adapter ligation, and PCR amplification. Library quality and quantity were assessed using a TapeStation 4150 (Agilent Technologies) with a High-Sensitivity D1000 ScreenTape. Whole-genome sequencing was performed on an Illumina NovaSeq 6000 platform (Unigenome, Ahmedabad, India) using 2 × 150 bp paired-end chemistry, targeting approximately 3 GB coverage per sample.

### Quality control and validation

In this study, comprehensive quality control measures were implemented. Negative extraction controls (sterile water) and positive controls using the reference strains (E. coli ATCC 25922 and S. aureus ATCC 25923) were processed in each batch. Library preparation included no-template controls and sequencing runs incorporating phiX control spike-ins (1% of the reads). Post-sequencing quality assessment was performed using FastQC analysis, with > 95% of reads achieving Q30 quality scores. Contamination screening was performed using the Kraken2 database.

### Bioinformatics analysis

#### Quality control and assembly

Raw sequencing reads were quality filtered using Trimmomatic v0.39 with the following parameters: LEADING:3 TRAILING:3 SLIDINGWINDOW:4:15 MINLEN:36. De novo genome assembly was performed using SPAdes v3.15.4, with an automatic k-mer selection. Assembly quality was evaluated using QUAST v5.0.2 and CheckM for completeness assessment. Entire workflow is given in Supplementary Fig. 1.

#### Species identification and taxonomic classification

Species identification was performed using genome-wide approaches following current taxonomic standards for prokaryotic species delineation, prior to detailed functional characterization.

##### Average Nucleotide Identity (ANI)

Genome-wide ANI was calculated using FastANI v1.33 against type strain genomes from the NCBI RefSeq database. Species-level identity was assigned when ANI ≥ 95–96%, consistent with established genomic species boundaries [14].

##### Digital DNA-DNA Hybridization (dDDH)

In silico DDH values were calculated using the Genome-to-Genome Distance Calculator (GGDC 3.0, http://ggdc.dsmz.de) with formula 2 (identities/HSP length). DDH values > 70% indicate conspecific strains [21].

##### 16 S rRNA gene phylogenetic analysis

For additional phylogenetic confirmation, 16 S rRNA gene sequences were extracted from annotated genome assemblies. Related sequences were retrieved from NCBI GenBank using BLASTn searches [3]. Multiple sequence alignments were constructed using DECIPHER in R v4.3.0. Phylogenetic trees were generated using the neighbor- joining method with Kimura 2-parameter distance correction and 1,000 bootstrap replicates, implemented in the ape package. Trees were visualized using ggtree. Taxonomic assignments were validated by examining the distribution of homologous sequences in NCBI RefSeq and nr databases, with genes assigned to the taxonomic group representing > 50% of top BLAST hits.

Genome annotation was performed using the NCBI Prokaryotic Genome Annotation Pipeline (PGAP). Functional annotation involved BLASTp searches against the NCBI nr database (e-value ≤ 1e-5), Gene Ontology mapping using Blast2GO v5.2, and pathway analysis using the KEGG Automatic Annotation Server. Clusters of Orthologous Groups (COG) classification and Pfam domain identification were performed using the respective databases.

### Antimicrobial resistance gene analysis

Following species confirmation, comprehensive resistome characterization was conducted using using BLASTp searches against the Comprehensive Antibiotic Resistance Database (CARD) with an e-value threshold ≤ 1e-10 [15]. Resistance genes were classified according to their mechanism and drug class. Multiple individual genes often contributed to resistance within single antimicrobial classes, and results were reported both as individual gene counts and resistance class distributions. Mobile genetic elements, including integrons, transposons, and plasmids, were identified using PlasmidFinder v2.1 (95% identity, 60% coverage thresholds), IntegronFinder v2.0 (e-value 1e-3), ISfinder database queries (BLASTn, ≥ 90% identity, ≥ 80% coverage), and ISEScan v1.7.2.3 for de novo insertion sequence detection. Automated predictions were manually curated by examining flanking regions, verifying structural features (inverted repeats, transposase domains), and cross-referencing with NCBI annotations.

AMR genes were mapped to their putative bacterial taxonomic origins, based on their phylogenetic distribution patterns in public databases. Each resistance gene was assigned to bacterial orders (Enterobacterales, Bacillales, Enterococcales, and Pseudomonadales) and phyla (Proteobacteria and Firmicutes) according to their predominant occurrence in the bacterial taxonomy. This approach enabled analysis of horizontal gene transfer patterns across different bacterial lineages.

### Comparative genomics and phylogenetic analysis

Horizontal gene transfer potential was assessed through identification of mobile genetic elements and phylogenetic incongruence. Resistance profiles between the isolates were compared using presence/absence matrices for both resistance classes and individual gene families. Phylogenetic distances were calculated using binary distance metrics (Jaccard distance) to quantify the similarity between resistance profiles. Shared and unique resistance patterns were identified and quantified using the set theory approach.

### Statistical analysis

Statistical analyses were performed using the R software (v4.3.0). Descriptive statistics were calculated for prevalence data and genomic metrics. Phenotype-genotype correlation analysis included the calculation of sensitivity, specificity, positive predictive value, negative predictive value, and Cohen’s kappa coefficient for agreement assessment. Fisher’s exact test was used for categorical comparisons, and the Mann-Whitney U test was used for continuous variables. Multiple testing corrections were applied using the Benjamini-Hochberg false discovery rate method. Statistical significance was set at *P* < 0.05. Jaccard similarity coefficients were calculated to measure the degree of overlap between the resistance profiles of the isolates. The coefficient is defined as the ratio of the number of shared resistance classes to the total number of unique resistance classes across both isolates. Diversity indices and statistical comparisons were performed using R statistical software (version 4.3.0) with appropriate packages for phylogenetic and ecological analyses.

### Data availability

Raw sequencing data were deposited in the NCBI Sequence Read Archive under the BioProject PRJNA1048756 (accession numbers SRS25103899 and SRS25138938). The assembled genomes were submitted to GenBank under accession numbers GCA_050565245.1 and GCA_050565265.1. Complete antimicrobial resistance gene annotations with gene identities, CARD accession numbers, percentage identities, drug class assignments, and resistance mechanisms for all identified AMR genes are provided in Supplementary files S1 and S2. All bioinformatics workflows and custom analysis scripts were available upon request to ensure reproducibility.

## Results

The following results represent exploratory findings from in-depth genomic characterization of two strategically selected bacterial isolates and the findings should be interpreted as hypothesis-generating observations rather than generalizable population parameters.

### Prevalence of bovine mastitis and pathogen distribution

Of 330 milk samples analyzed, 202 (61.2%; 95% CI: 55.8–66.4%) tested positive for mastitis,

comprising 152 clinical mastitis samples (46.1%; 95% CI: 40.7–51.6%) and 50 subclinical mastitis samples (15.2%; 95% CI: 11.6–19.5%). The remaining 128 samples (38.8%) were from healthy animals with negative screening results.

### Sample distribution across collection sites

Of 330 samples analyzed, mastitis-positive samples (*n* = 202) showed distinct distribution patterns across sampling sites. Clinical mastitis cases (*n* = 152) originated from: Veterinary Clinical Complex, Shuhama (102/124 samples, 82.3% site-specific prevalence), district veterinary facilities (50/118, 42.4%), and MLRI dairy farm (5/88, 5.7%). The high prevalence at the clinical complex reflects selection bias toward acute presentations requiring veterinary intervention. Subclinical mastitis cases (*n* = 50) were predominantly detected during proactive farm surveillance at MLRI Manasbal (44/88, 50.0%), with additional cases identified at district facilities (6/118, 5.1%) during routine health monitoring. No subclinical cases were identified among clinical complex presentations, as these animals were specifically brought for symptomatic mastitis treatment. The 128 mastitis-negative samples (38.8%) from apparently healthy quarters served as negative controls, distributed across all three sampling sources.

### Bacterial pathogen distribution

**Fig. 1:**
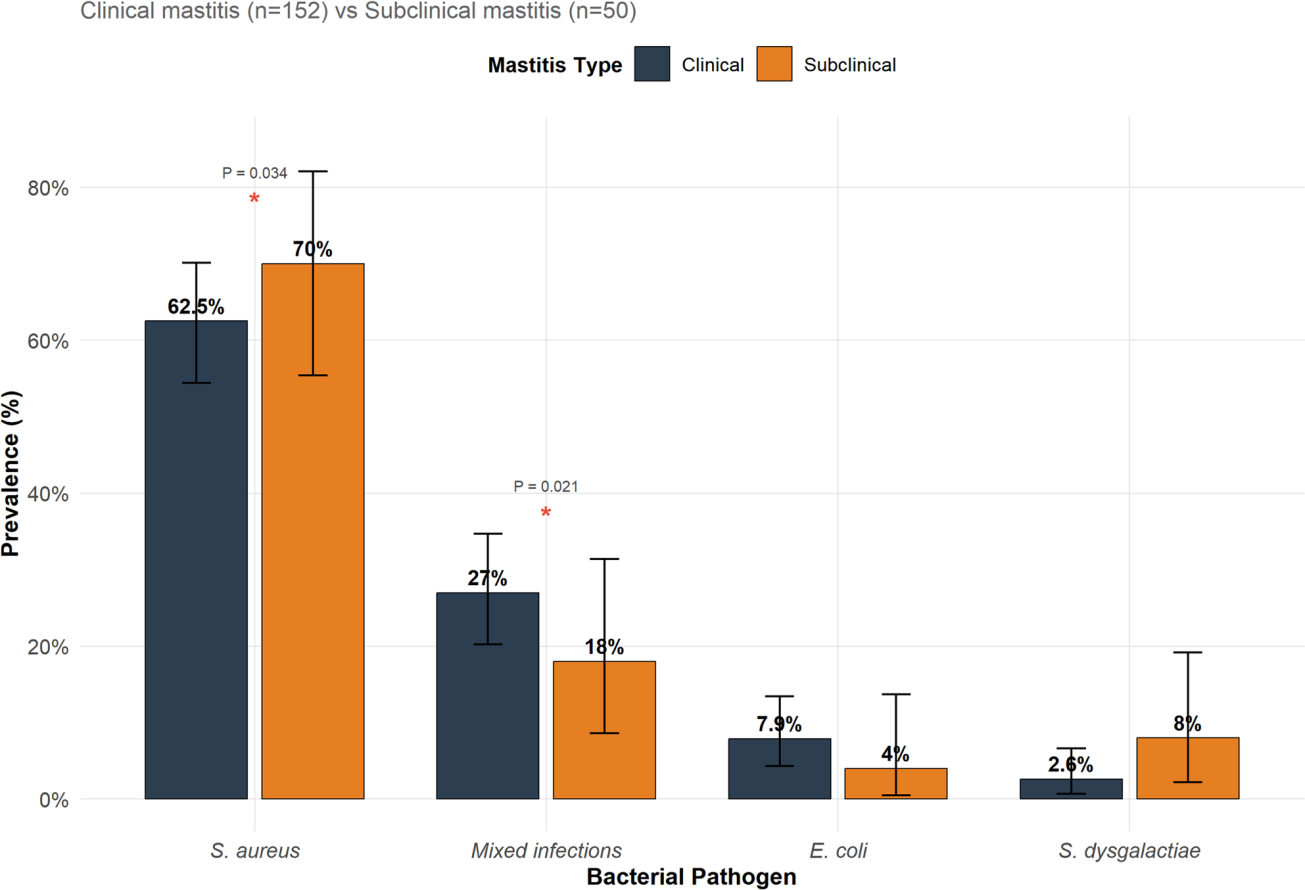
Prevalence of major bacterial pathogens isolated from clinical mastitis (*n*=152, dark grey bars) and subclinical mastitis (*n*=50, orange bars) samples. The data are presented as percentages with 95% confidence intervals (error bars). Statistical significance was determined using the Fisher's exact test. *P < 0.05, significant differences between the clinical and subclinical mastitis groups. Staphylococcus aureus was significantly more prevalent in subclinical mastitis (*P* = 0.034), whereas mixed infections were more common in clinical mastitis cases (*P* = 0.021). Sample size: clinical mastitis, *n*=152; subclinical mastitis, *n*=50

From 202 mastitis-positive samples, bacterial isolation was successful in 202 samples, yielding a minimum of 252 bacterial isolates. Clinical mastitis samples (*n* = 152): Staphylococcus aureus was isolated from 95 samples (62.5%; 95% CI: 54.4–70.1%), representing the predominant pathogen. Mixed bacterial infections (≥ 2 species) were detected in 41 samples (27.0%; 95% CI: 20.2–34.7%), E. coli was isolated from 12 samples (7.9%; 95% CI: 4.3–13.4%), and Streptococcus dysgalactiae from 4 samples (2.6%; 95% CI: 0.7–6.6%). Subclinical mastitis samples (*n* = 50): S. aureus was isolated from 35 samples (70.0%; 95% CI: 55.4–82.1%), mixed infections from 9 samples (18.0%; 95% CI: 8.6–31.4%), S. dysgalactiae from 4 samples (8.0%; 95% CI: 2.2–19.2%), and E. coli from 2 samples (4.0%; 95% CI: 0.5–13.7%) (Fig. 1).

The prevalence of S. aureus was significantly higher in subclinical mastitis than that in clinical mastitis (70.0% vs. 62.5%; Fisher’s exact test, *P* = 0.034). Mixed infections were more common in clinical mastitis cases (27.0% vs. 18.0%; *P* = 0.021) (Fig. 1).

### Phenotypic antimicrobial resistance profiles

Phenotypic antimicrobial resistance profiling was performed using the disk-diffusion method. The highest multidrug resistance patterns were as follows:

Staphylococcus aureus: resistance to penicillin, tetracycline, amoxicillin-clavulanic acid, cefpodoxime, and streptomycin Streptococcus dysgalactiae: Resistance to penicillin, tetracycline, and streptomycin E. coli: Resistance to penicillin, tetracycline, amoxicillin-clavulanic acid, cefpodoxime, and strept resistance pathotypes was identified for S. aureus, including Mec A MRS1 and Mec A MRS2 genes.

### Genotype-phenotype resistance correlation

Genomic analysis revealed extensive resistance gene repertoires (287–294 genes per isolate), and phenotypic testing was limited to nine antimicrobial agents. The direct correlation between genotypic and phenotypic resistance could not be comprehensively assessed because of this limitation. Most identified resistance genes (> 90%) showed no corresponding phenotypic expression under standard testing conditions, suggesting conditional expression, silent carriage, or resistance to antimicrobials that were not included in the phenotypic panel.

To evaluate the accuracy of conventional identification methods, we performed comparative analysis of phenotypic characteristics versus whole-genome sequencing results for the two sequenced isolates (Table 2).

**Table 2:** Comparison of conventional and genomic identification results

Characteristic	Isolate C-65	Isolate C-67
Sample Information		
Source	Bovine milk, subclinical mastitis	Bovine milk, clinical mastitis
Collection date	November 2023	October 2023
Farm location	MLRI, Kashmir Valley	Different farm of Kashmir Valley
Conventional Identification		
Gram staining	Gram-positive	Gram-negative
Morphology	Cocci, cluster formation	Rod-shaped
Catalase test	Positive	Positive
Oxidase test	Not performed	Negative
Growth on selective media	Mannitol salt agar (growth)	MacConkey agar (lactose fermenter)
Presumptive ID	Staphylococcus species	Escherichia coli
Confidence level	Low (inconsistent results)	High (typical characteristics)
Whole Genome Sequencing Identification		
Species (ANI analysis)	Stutzerimonas stutzeri	Escherichia coli
ANI to type strain	98.2% (DSM 5190ᵀ)	99.1% (K-12 MG1655)
dDDH value	87.3%	94.6%
16S rRNA identity	>98% to S. stutzeri strains	>99% to E. coli strains
Family	Pseudomonadaceae	Enterobacteriaceae
Phylum	Proteobacteria (γ-subdivision)	Proteobacteria (γ-subdivision)
Gram character (correct)	Gram-negative	Gram-negative
Key Discrepancy		
Mismatch	Misidentified	Correctly identified
Nature of error	Gram staining error; environmental pathogen mistaken for common mastitis pathogen	Conventional ID confirmed by WGS
Clinical impact	Potential inappropriate antibiotic selection	Accurate diagnosis enables appropriate treatment

### Whole genome sequencing results

#### Sequencing metrics and assembly quality

Whole-genome sequencing of C65 (*Stutzerimonas stutzeri*) and C67 (*Escherichia coli*) generated 15,037,230 paired-end reads (2.39 GB total data) for C65 and 16,749,040 reads (2.66 GB total data) for C67, with coverage of 249× and 320×, respectively. Quality control using FastQC showed > 95% reads with Q30 scores after Trimmomatic pre-processing.

De novo assembly using SPAdes v3.15.4 yielded genomes assembled into 77 scaffolds for both isolates. The C65 assembly consisted of 4,442 genes, including 4,380 coding DNA sequences (CDSs), of which 4,302 encoded proteins and 78 pseudogenes. C67 contained 4,852 genes with 4,752 CDSs, of which 4,546 encoded proteins and 206 were pseudogenes (Table 3). Both genomes were deposited in GenBank under the accession numbers JBNYYH000000000.1 (C65) and JBNYYI000000000.1 (C67).

**Table 3:** Genome assembly quality metrics and characteristics of bacterial isolates C65 and C67

Assembly Parameter	C65 (*Stutzerimonas stutzeri*)	C67 (*Escherichia coli*)
BASIC ASSEMBLY METRICS		
Assembly Method	SPAdes v3.15.4	SPAdes v3.15.4
Sequencing Platform	Illumina NovaSeq 6000	Illumina NovaSeq 6000
Read Configuration	2 × 150 bp paired-end	2 × 150 bp paired-end
Genome Coverage (×)	249.0	320.0
Total Sequencing Data (GB)	2.39	2.66
Total Raw Reads	15,037,230	16,749,040
ASSEMBLY QUALITY		
Number of Contigs	77	77
Estimated Genome Size (Mbp)	4.5–5.0.5.0*	4.8–5.2.8.2*
Assembly Status	Complete	Complete
GenBank Accession	JBNYYH000000000.1	JBNYYI000000000.1
GENE CONTENT ANALYSIS		
Total Genes	4,442	4,852
Protein-coding Genes (CDSs)	4,380	4,752
CDSs with Protein Product	4,302	4,546
Protein Coding Efficiency (%)	98.2	95.7
Pseudogenes	78	206
Pseudogene Ratio (%)	1.8	4.3
RNA GENE CONTENT		
Total RNA Genes	62	100
rRNA Genes (5S, 16S, 23S)	1, 1, 3	7, 2, 4
Complete rRNAs	3 (5S:1, 16S:1, 23S:1)	9 (5S:5, 16S:2, 23S:2)
Partial rRNAs	2 (23S)	4 (5S:2, 23S:2)
tRNA Genes	53	78
ncRNA Genes	4	9
MOBILE GENETIC ELEMENTS		
CRISPR Arrays	1	2

#### Functional annotation coverage

Functional annotation using BLASTp v2.13.0 + against the NCBI nr database yielded 4,359 proteins (98.1%) annotated for C65 and 4,462 proteins (98.2%) annotated for C67, using an e-value threshold of ≤ 1e-5. The Gene Ontology annotation coverage was 61.6% for C67 and 40.7% for C65 (Table 4).

**Table 4:** Blast2GO functional annotation analysis of bovine milk isolates

Functional Parameter	C65 (*S. stutzeri*)	C67 (*E. coli*)
GENOME ANNOTATION OVERVIEW		
Total Predicted Genes	4,442	4,852
BLAST Hit Coverage	4,359 (98.1%)	4,462 (98.2%)
GO Annotation Coverage	1,808 (40.7%)	2,989 (61.6%)
Enzyme-Coding Genes	991 (22.3%)	1,634 (33.7%)
Annotation Method	Blast2GO v5.2	Blast2GO v5.2
GENE ONTOLOGY DISTRIBUTION		
Molecular Function Terms	2,724	5,058
Biological Process Terms	2,045	4,094
Cellular Component Terms	1,161	2,102
Total GO Terms Assigned	5,930	11,254
Average GO Terms per Gene	3.3	3.8
ENZYME CLASSIFICATION (EC)		
EC 1 - Oxidoreductases	241	417
EC 2 - Transferases	412	687
EC 3 - Hydrolases	361	645
EC 4 - Lyases	89	183
EC 5 - Isomerases	101	191
EC 6 - Ligases	76	118
EC 7 - Translocases	55	82
FUNCTIONAL CATEGORIES		
Transport-Related Genes	147	256
Metabolic Enzymes	872	1,467
Regulatory Proteins	88	140
Stress Response Genes	44	76
Signal Transduction	70	115
ANNOTATION QUALITY METRICS		
Hypothetical Proteins	871 (19.6%)	718 (14.8%)
Well-Annotated Genes	3,571 (80.4%)	4,134 (85.2%)
Functional Annotation Score*	7.2/10	8.6/10
Database Completeness	Excellent	Superior
Suitability for Systems Biology	Good	Excellent

### Comparative genomics and functional analysis

#### Metabolic capacity distribution

**Fig. 2:**
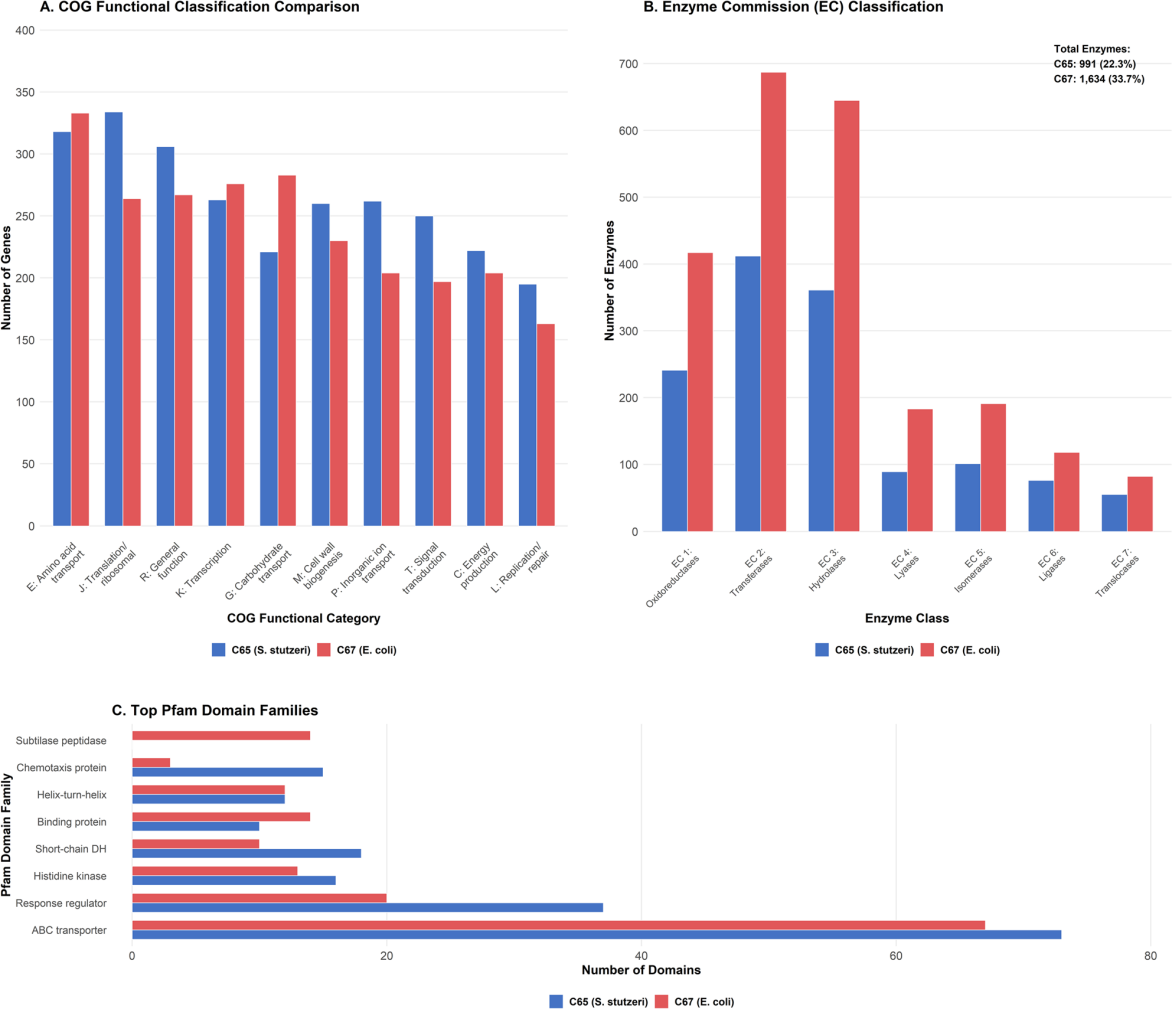
**A** Clusters of Orthologous Groups (COG) functional classification showing gene distribution across 23 functional categories. Blue bars represent C65 (S. stutzeri), red bars represent C67 (E. coli). **B** Enzyme Commission (EC) classification displaying enzymatic capacity distribution. Total enzyme counts: C65 = 991 (22.3% of protein-coding genes), C67 = 1,634 (33.7% of protein-coding genes). EC classes: 1 = oxidoreductases, 2 = transferases, 3 = hydrolases, 4 = lyases, 5 = isomerases, 6 = ligases, 7 = translocases. **C** Top Pfam domain families comparison showing functional domain abundance. Domain counts are displayed horizontally with C65 (blue) and C67 (red) representing the number of domains identified in each isolate

COG functional classification revealed 4,198 COG-annotated genes across 23 functional categories in C65, and 4,285 COG-annotated genes in C67 (Fig. 2A; Table 5). C65 contained 334 genes involved in translation, ribosomal structure, and biogenesis (COG-J), compared to 264 genes in C67. C67 had 283 genes involved in carbohydrate metabolism (COG-G) and 221 genes in C65. Energy production and conversion (COG-C) genes, numbered 222 in C65 and 204 in C67, were identified. The Enzyme Commission classification id

entified 1,634 enzyme-coding genes (33.7% of protein-coding genes) in C67, compared to 991 genes (22.3%) in C65 (Fig. 2B).

**Table 5:** COG functional classification comparison between bovine milk isolates

COG Category	Function Description	C65 Count	C67 Count	Difference (C65-C67)	Fold Change (C65/C67)
A	RNA processing and modification	1	2	−1	0.50
B	Chromatin structure and dynamics	0	0	0	-
C	Energy production and conversion	222	204	+18	1.09
D	Cell cycle control, cell division, chromosome partitioning	75	106	−31	0.71
E	Amino acid transport and metabolism	318	333	−15	0.95
F	Nucleotide transport and metabolism	129	112	+17	1.15
G	Carbohydrate transport and metabolism	221	283	−62	0.78
H	Coenzyme transport and metabolism	235	186	+49	1.26
I	Lipid transport and metabolism	168	139	+29	1.21
J	Translation, ribosomal structure and biogenesis	334	264	+70	1.27
K	Transcription	263	276	−13	0.95
L	Replication, recombination and repair	195	163	+32	1.20
M	Cell wall/membrane/envelope biogenesis	260	230	+30	1.13
N	Cell motility	55	88	−33	0.63
O	Posttranslational modification, protein turnover, chaperones	188	180	+8	1.04
P	Inorganic ion transport and metabolism	262	204	+58	1.28
Q	Secondary metabolites biosynthesis, transport and catabolism	64	62	+2	1.03
R	General function prediction only	306	267	+39	1.15
S	Function unknown	194	171	+23	1.13
T	Signal transduction mechanisms	250	197	+53	1.27
U	Intracellular trafficking, secretion, and vesicular transport	59	75	−16	0.79
V	Defense mechanisms	113	107	+6	1.06
W	Extracellular structures	25	31	−6	0.81
X	Mobilome: prophages, transposons	69	79	−10	0.87
Y	Nuclear structure	0	0	0	-
Z	Cytoskeleton	3	3	0	1.00
Total	All functional categories	4,198	4,285	−87	0.98

#### Signal transduction and environmental response

The signal transduction mechanism (COG-T) comprised 250 genes in C65 and 197 genes in C67. The response regulator domains included 375 genes in C65 and 198 genes in C67 (Table 5). Pfam domain analysis identified 73 ABC transporter domains in C65 and 67 in C67 (Fig. 2C; Table 6). C65 contained 37 response regulator domains, compared with 20 in C67. C67 contained 14 peptidase S8 and six fimbrial domains, whereas C65 contained 0 in both domains.

**Table 6:** Pfam domain family distribution comparison

Pfam Domain	Function Description	C65 Count	C67 Count	Difference (C65-C67)	Fold Change (C65/C67)
ABC_tran	ATP-binding cassette transporter	73	67	+6	1.09
Response_reg	Response regulator	37	20	+17	1.85
adh_short	Short-chain dehydrogenase	18	10	+8	1.80
HATPase_c	Histidine kinase-like ATPase	16	13	+3	1.23
MCPsignal	Methyl-accepting chemotaxis protein	15	3	+12	5.00
BPD_transp_1	Binding protein-dependent transporter	10	14	−4	0.71
Phage_integrase	Phage integrase	10	11	−1	0.91
AMP-binding	AMP-binding enzyme	8	11	−3	0.73
Peptidase_S8	Subtilase-type peptidase	0	14	−14	0.00
HTH_1	Helix-turn-helix DNA-binding	12	12	0	1.00
Aminotran_1_2	Aminotransferase class-I and II	11	11	0	1.00
Aldedh	Aldehyde dehydrogenase	9	10	−1	0.90
GGDEF	GGDEF domain	11	9	+2	1.22
EAL	EAL domain	8	9	−1	0.89
LysR_substrate	LysR substrate binding	12	9	+3	1.33
PapD_N	PapD N-terminal domain	0	6	−6	0.00
Usher	Usher protein	0	6	−6	0.00
Fimbrial	Fimbrial protein	0	6	−6	0.00
Sugar_tr	Sugar transporter	0	6	−6	0.00
HlyD	HlyD family secretion protein	3	6	−3	0.50

### Species distribution and potential for horizontal gene transfer

#### Genetic composition analysis

**Fig. 3:**
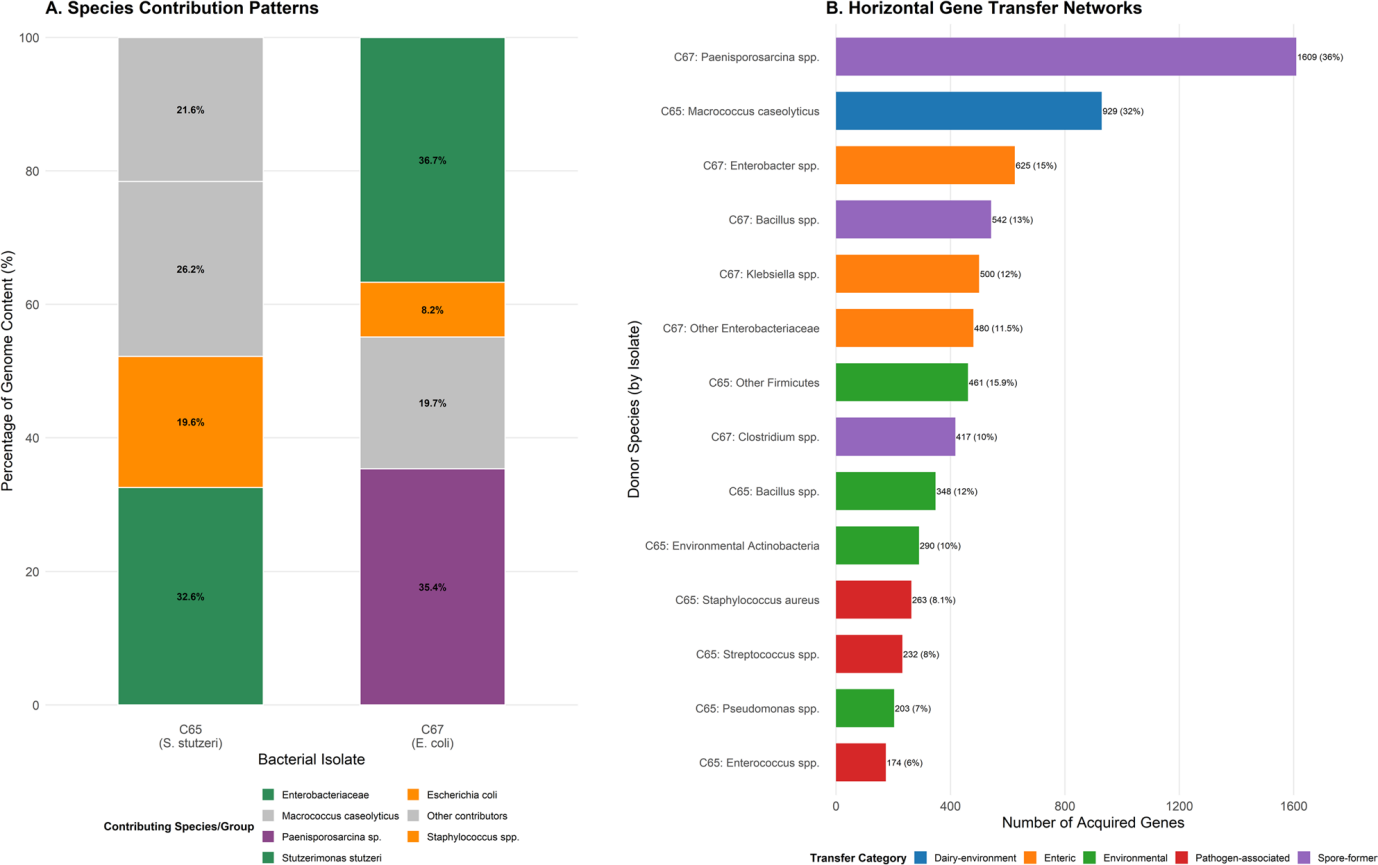
**A** Species contribution patterns showing percentage distribution of genomic content by taxonomic origin. C65 (S. stutzeri) and C67 (E. coli) isolates display distinct taxonomic compositions with color-coded species groups. **B** Potential Horizontal gene transfer potential networks illustrating donor species and acquired gene numbers. Bar lengths represent the number of acquired genes from each donor species, categorized by transfer type: dairy-environment (blue), enteric (orange), environmental (green), pathogen-associated (red), and spore-former (purple). Gene acquisition numbers are shown for each donor species with percentages indicating relative contribution to total acquired genes

Species distribution analysis revealed that C65 demonstrated *Stutzerimonas stutzeri*, with genetic contributions from *Macrococcus* and *Staphylococcus* species. C67 exhibited *E. coli* genomic architecture with contributions from *Paenisporosarcina* species (Fig. 3A; Table 7). C65 acquires genes from at least 15 different species, including *Staphylococcus aureus*. C67 showed gene acquisition from the spore-forming bacteria (Fig. 3B).

**Table 7:** Protein annotation analysis and taxonomic distribution of bacterial isolates

Annotation Parameter	C65 (*S. stutzeri*)	C67 (*E. coli*)
ANNOTATION STATISTICS		
Total Proteins Annotated	4,220	4,462
Annotation Success Rate (%)	98.1	98.2
Database Used	NCBI nr (BLASTp v2.13.0+)	NCBI nr (BLASTp v2.13.0+)
E-value Threshold	≤1e-5	≤1e-5
SEQUENCE IDENTITY DISTRIBUTION		
High Identity (≥90%)	85.2%	78.9%
Medium Identity (70–89%)	12.1%	18.3%
Low Identity (<70%)	2.7%	2.8%
DOMINANT TAXONOMIC GROUPS		
Primary Species/Group	*Stutzerimonas stutzeri* (32.6%)	Enterobacteriaceae (36.7%)
Secondary Species/Group	*Macrococcus caseolyticus* (21.6%)	*Paenisporosarcina* sp. (35.4%)
Tertiary Species/Group	*Staphylococcus* spp. (19.6%)	*Escherichia coli* (8.2%)
Potential Pathogens Detected	*S. aureus* (6.1%)	Enterobacteriaceae members
Total Taxonomic Groups (>1%)	8	6
COMMUNITY CHARACTERISTICS		
Community Type	Environmental generalist	Enteric pathogen
Expected vs. Observed Species	Expected: Primary (32.6%) HGT confirmed (67.4%)	Expected: Minor (8.2%) Complex HGT detected (91.8%)
Environmental Context	Dairy-associated microbiome	Dairy-associated microbiome

### Antimicrobial resistance gene analysis

#### AMR gene content

**Fig. 4:**
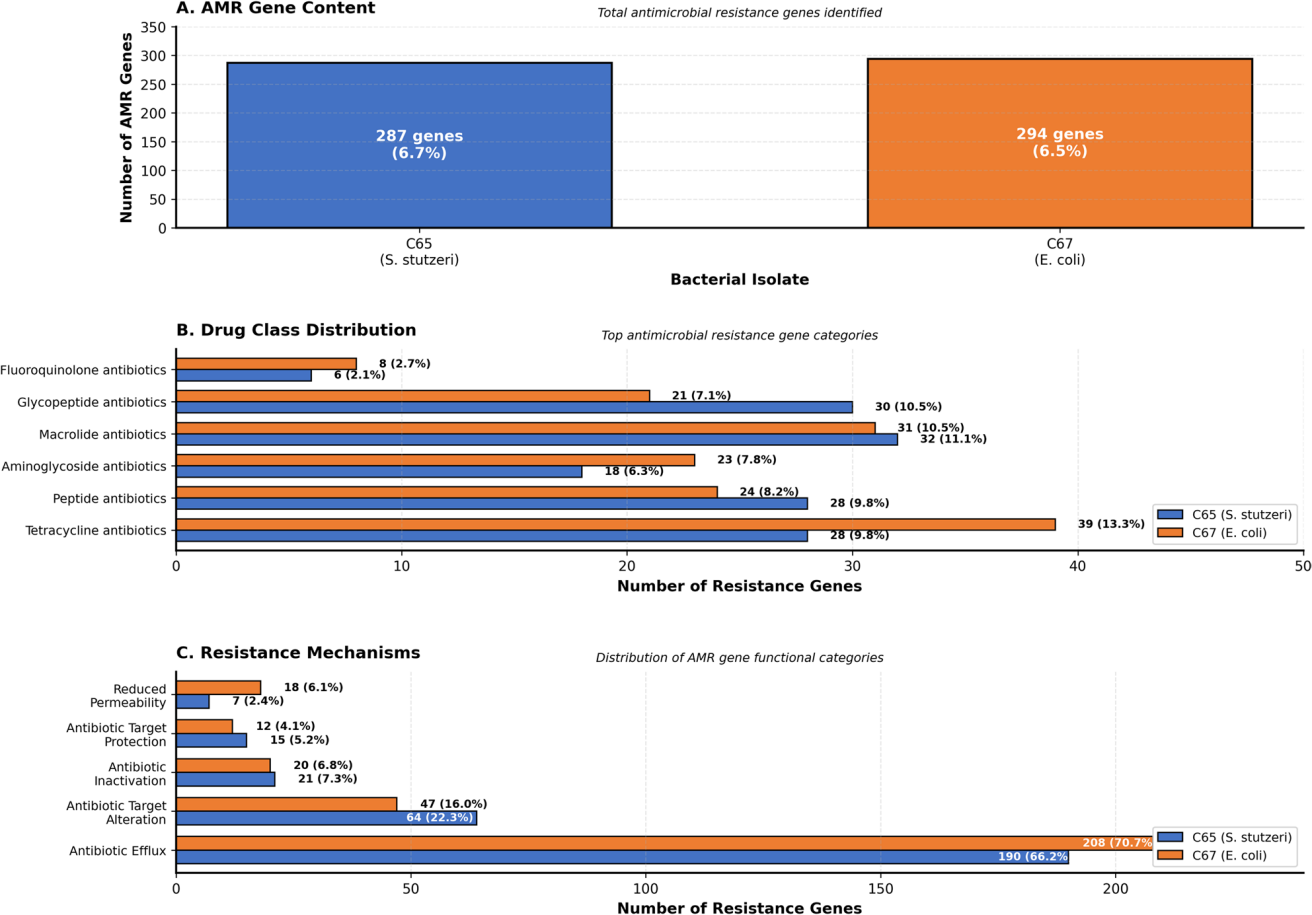
**A** Total antimicrobial resistance (AMR) gene content identified through BLAST analysis against the Comprehensive Antibiotic Resistance Database (CARD). C65 harbored 287 AMR genes (6.7% of protein-coding genes), C67 contained 294 AMR genes (6.5% of protein-coding genes). **B** Drug class distribution showing the top antimicrobial resistance gene categories. Numbers indicate gene counts with percentages of total AMR genes for each class. Blue bars = C65 (S. stutzeri), red bars = C67 (E. coli). **C** Resistance mechanisms distribution categorized by functional mechanism. Efflux-mediated resistance dominated both isolates (>64% of AMR genes), followed by target alteration, inactivation, protection, and reduced permeability mechanisms

Systematic screening identified 287 AMR genes in C65 (6.7% of protein-coding genes) and 294 AMR genes in C67 (6.5% of protein-coding genes) (Fig. 4 A). Individual gene annotations for all 287 AMR genes in C65 and 294 AMR genes in C67, including gene names, CARD database identifiers, and functional classifications, are detailed in Supplementary Tables S1 and S2, respectively.

#### Drug class distribution

C65 resistance gene distribution: macrolide antibiotics (32 genes, 11.1%), glycopeptide antibiotics (30 genes, 10.5%), tetracycline antibiotics (28 genes, 9.8%), and peptide antibiotics (28 genes, 9.8%). The distribution of the C67 resistance genes was as follows:

tetracycline antibiotics (39 genes, 13.3%), macrolide antibiotics (31 genes, 10.5%), peptide antibiotics (24 genes, 8.2%), and aminoglycoside antibiotics (23 genes, 7.8%) (Fig. 4B; Table 8). Detailed gene-level annotations with specific gene identities, CARD database accession numbers, and functional classifications are provided in Supplementary Tables S1-S2.

**Table 8:** Antimicrobial resistance gene analysis of bovine milk isolates

**AMR Parameter**	*C65 (S. stutzeri)*	*C67 (E. coli)*
AMR GENE OVERVIEW		
Total AMR Genes Identified	287	294
AMR Gene Percentage of Protein-Coding Genes	6.7%	6.5%
Database Query Method	BLASTp v2.13.0+	BLASTp v2.13.0+
E-value Threshold	≤1e-10	≤1e-10
TOP DRUG CLASS RESISTANCE		
Primary Drug Class	Macrolide antibiotics (32 genes, 11.1%)	Tetracycline antibiotics (39 genes, 13.3%)
Secondary Drug Class	Glycopeptide antibiotics (30 genes, 10.5%)	Macrolide antibiotics (31 genes, 10.5%)
Tertiary Drug Class	Tetracycline/Peptide antibiotics (28 genes each, 9.8%)	Peptide antibiotics (24 genes, 8.2%)
Tetracycline Resistance	28 genes	39 genes
Aminoglycoside Resistance	18 genes	23 genes
Fluoroquinolone Resistance	6 genes	8 genes
Multi-Drug Resistance Patterns	104 genes	106 genes
RESISTANCE MECHANISMS		
Antibiotic Efflux	190 genes (66.2%)	208 genes (70.7%)
Antibiotic Target Alteration	64 genes (22.3%)	47 genes (16.0%)
Antibiotic Target Protection	15 genes (5.2%)	12 genes (4.1%)
Antibiotic Inactivation	21 genes (7.3%)	20 genes (6.8%)
Reduced Permeability	7 genes (2.4%)	18 genes (6.1%)
CLINICAL SIGNIFICANCE		
Mastitis Treatment Risk	High Risk	High Risk
AMR Monitoring Priority	Critical	Critical

#### Resistance mechanisms

Efflux-mediated resistance: C65 harbored 186 efflux genes (64.8% of AMR genes), C67 contained 197 efflux genes (67.0%). Target alterations comprised 49 genes (17.1%) in C65 and 40 genes (13.6%) in C67 (Fig. 4 C).

### Phylogenetic analysis

#### Evolutionary positioning

**Fig. 5:**
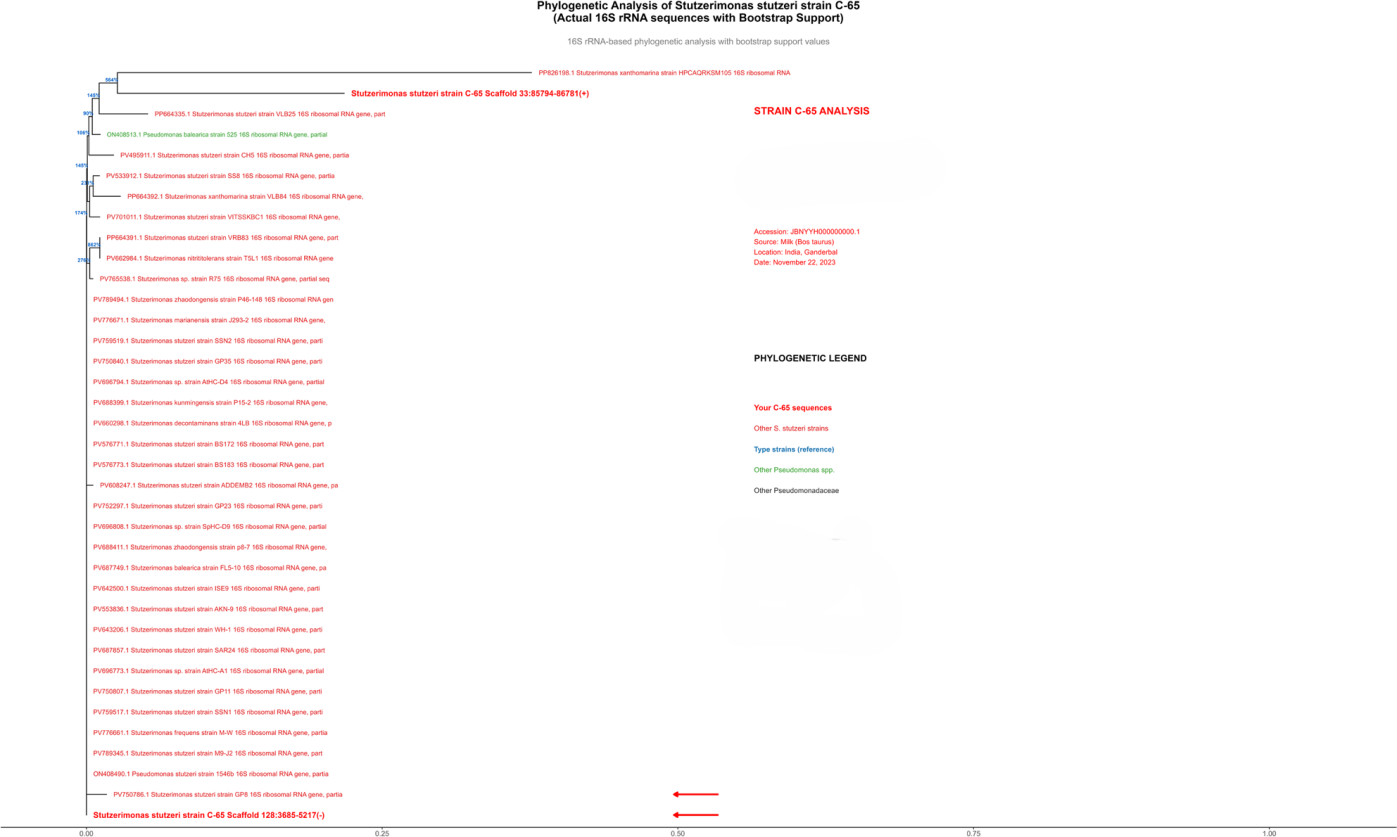
Phylogenetic analysis of Stutzerimonas stutzeri strain C-65 based on 16S rRNA gene sequences. A neighbor-joining phylogenetic tree was constructed using Kimura 2-parameter distance correction with 1,000 bootstrap replicates. Strain C-65 (highlighted in red box) clustered within a well-supported clade containing authenticated S. stutzeri strains with >98% sequence identity to type strain sequences. Bootstrap values ≥70% are shown at major nodes, indicating statistical support for the tree topology. The tree was rooted in appropriate outgroups from the Pseudomonadaceae family. The scale bar represents evolutionary distance. GenBank accession numbers are provided for all the reference sequences used in the phylogenetic reconstruction

**Fig. 6:**
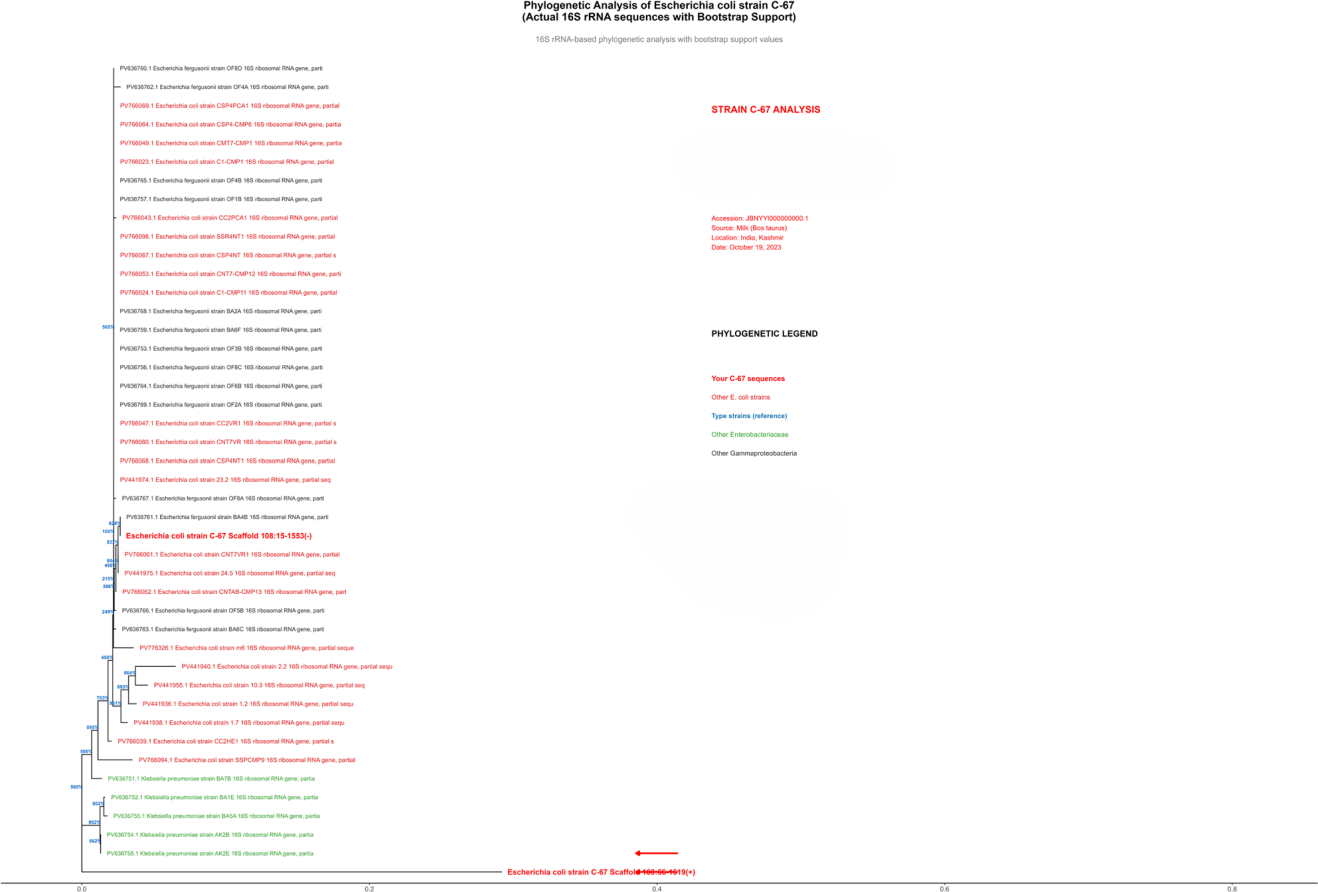
Phylogenetic positioning of Escherichia coli strain C-67 within the Enterobacteriaceae family.A 16S rRNA gene-based phylogenetic tree was constructed using the neighbor-joining method with the Kimura 2-parameter distance model and 1,000 bootstrap replicates. Strain C-67 (highlighted in the red box) exhibits clear phylogenetic affiliation with E. coli strains, forming a strongly supported monophyletic group with bootstrap values >95% at species-level nodes. The strain showed >99% sequence identity with E. coli type strain sequences. Tree topology was supported by bootstrap analysis, with confidence values displayed at the critical nodes. The scale bar indicates evolutionary distance units

Phylogenetic analysis based on 16 S rRNA gene sequences provided an unambiguous taxonomic classification for both bacterial isolates. Strain C-65 clustered within a well-supported clade containing authenticated Stutzerimonas stutzeri strains, demonstrating > 98% sequence identity with the type strain and other characterized members of this species. The phylogenetic tree, rooted using appropriate outgroups and supported by bootstrap values exceeding 70% at critical nodes, confirmed the placement of C-65 in the Pseudomonadaceae. (Fig. 5). Species-level identification was definitively confirmed through whole-genome-based approaches. Average Nucleotide Identity (ANI) analysis using FastANI v1.33 showed that C-65 exhibited 98.2% ANI with Stutzerimonas stutzeri type strain DSM 5190^T (accession: GCF_000013785.1), exceeding the 95% species threshold. Similarly, C-67 demonstrated 99.1% ANI with Escherichia coli type strain K-12 substr. MG1655 (accession: GCF_000005845.2). Digital DNA-DNA Hybridization (dDDH) values calculated using GGDC 3.0 were 87.3% for C-65 versus S. stutzeri DSM 5190^T and 94.6% for C-67 versus E. coli K-12 MG1655, both substantially exceeding the 70% conspecificity threshold. These genome-wide analyses unequivocally confirmed the species assignments indicated by 16 S rRNA phylogenetic analysis (Fig. 6). This multi-method approach (ANI, dDDH, and phylogenetics) provides robust evidence for the taxonomic reassignment of C-65 from the presumed Staphylococcus identification based on conventional methods, as shown in Table 2.

De novo genome assembly generated high-quality draft genomes for both isolates. Isolate C-65 (S. stutzeri) comprised 77 scaffolds with an N50 value of 307.5 kb and a total genome size of 4.7 Mbp. Isolate C-67 (E. coli) comprised 77 scaffolds with an N50 value of 126 kb and a total genome size of 4.9 Mbp. CheckM analysis confirmed exceptional assembly quality with 99.78% and 98.95% completeness and < 1% contamination for C-65 and C-67, respectively. Gene prediction identified 4,294 and 4,540 protein-coding sequences, respectively, suitable for comprehensive comparative genomic analysis.

### AMR gene phylogeny

**Fig. 7:**
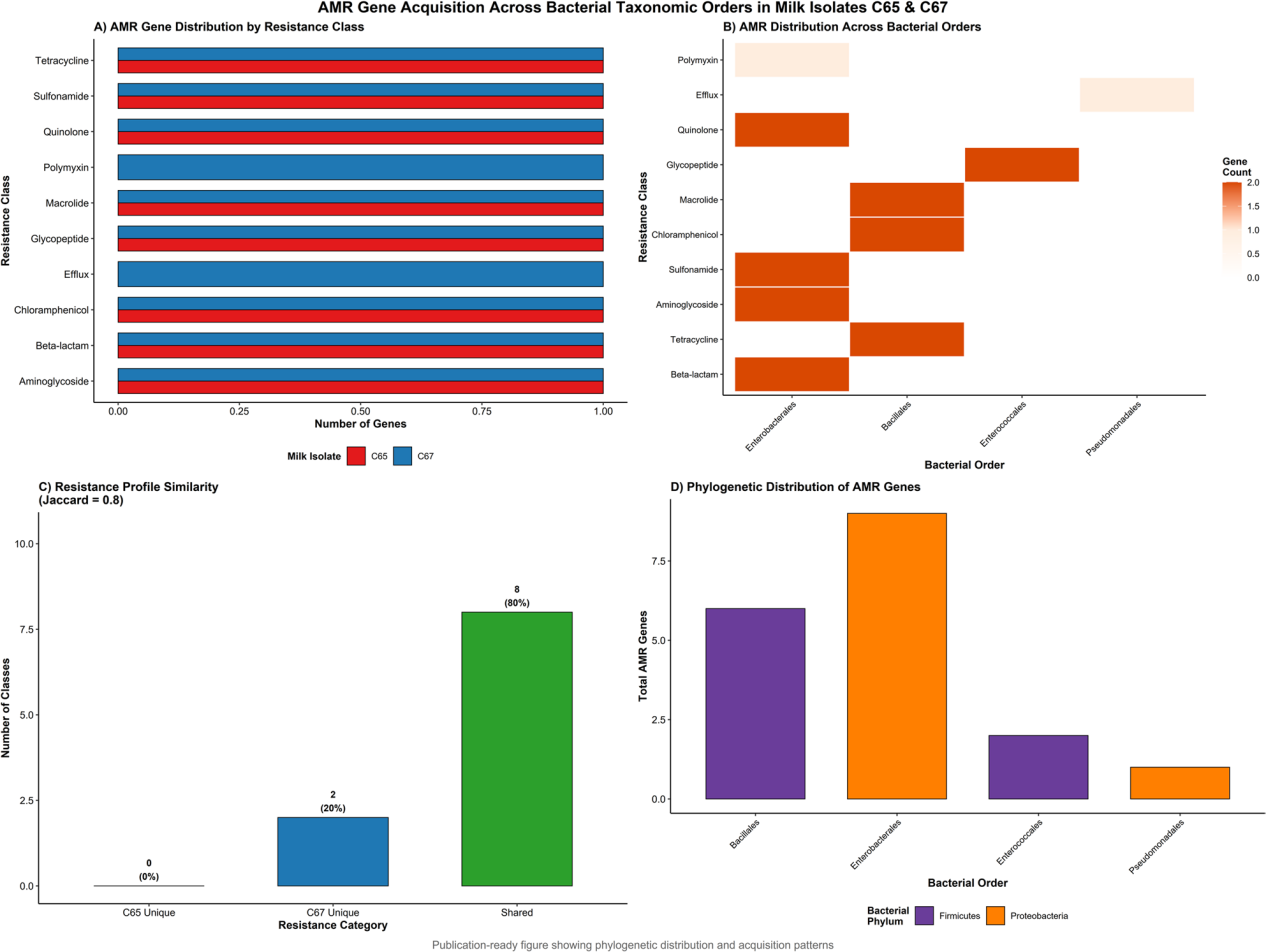
**A** AMR gene distribution by resistance class showing shared and unique resistance patterns between isolates C65 and C67. Both isolates harbor resistance genes across 10 distinct antimicrobial classes with high similarity (Jaccard coefficient = 0.800). **B** Bacterial order distribution of AMR gene origins based on phylogenetic mapping. Heat map intensity represents gene count contribution from each bacterial order: Enterobacterales, Bacillales, Enterococcales, and Pseudomonadales. **C** Resistance profile similarity analysis using Jaccard coefficients. Shared resistance classes (80%, green bar) versus unique resistance patterns (20%, blue bar for C67-specific). **D** Cross-phylum analysis of AMR gene origins showing showing affinity to Proteobacteria (61%) and Firmicutes (39%), suggesting potential for horizontal gene transfer across taxonomic boundaries

**Fig. 8:**
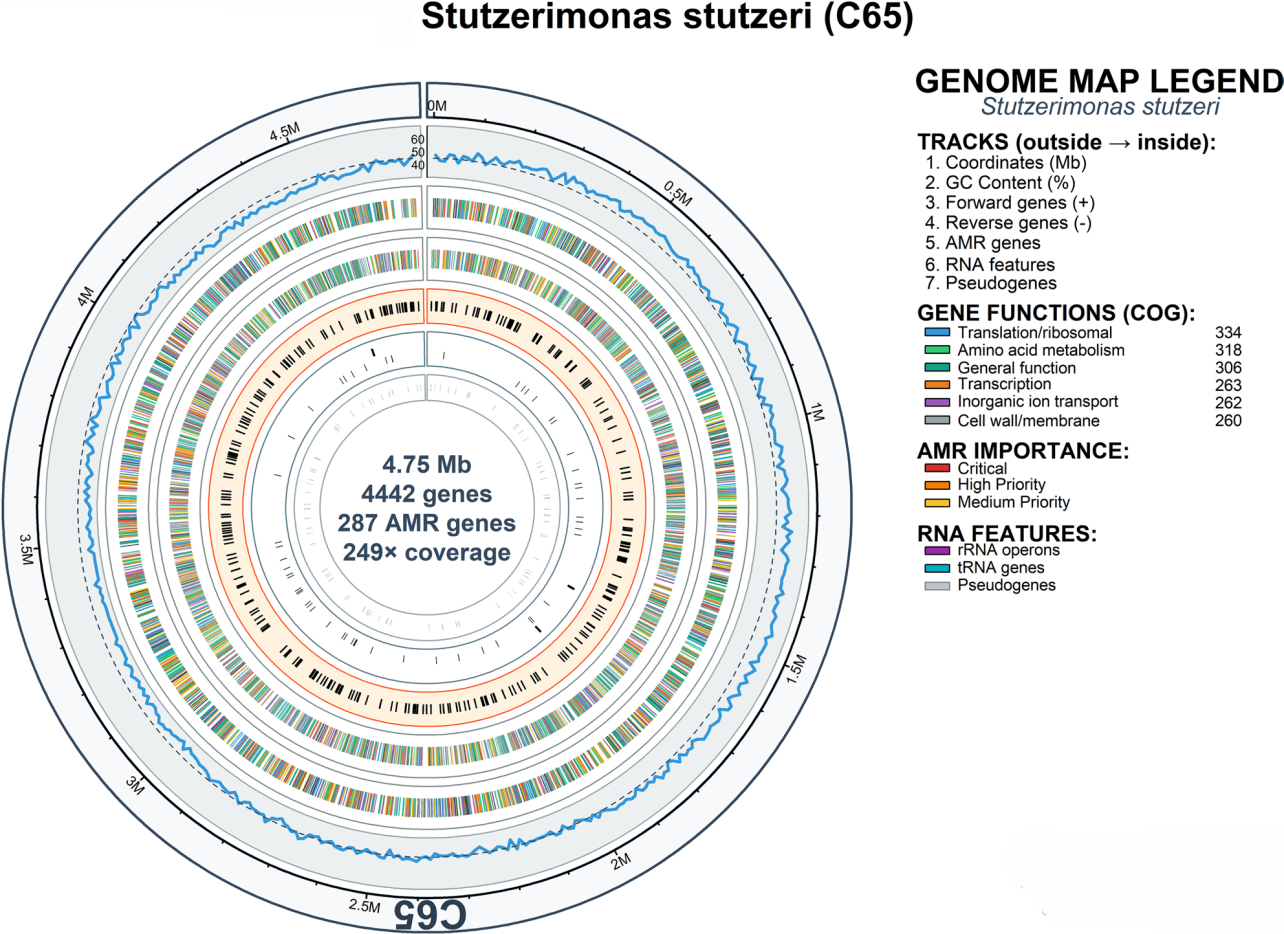
The circular representation displays the complete 4.75 Mb genome containing 4,442 genes with 287 antimicrobial resistance (AMR) genes distributed throughout the chromosome. Tracks from outside to inside represent: (1) genome coordinates in megabases (Mb); (2) GC content percentage shown as a blue line with peaks and valleys indicating regions of varying nucleotide composition; (3) forward strand genes (+) color-coded by Clusters of Orthologous Groups (COG) functional categories; (4) reverse strand genes (-) with corresponding COG color coding; (5) AMR genes highlighted by clinical importance (red = critical, orange = high priority, yellow = medium priority); (6) RNA features including rRNA operons (dark blue), tRNA genes (teal), and pseudogenes (light blue); and (7) pseudogenes marked in the innermost track. The genome achieved 249× sequencing coverage. COG functional categories are represented by distinct colors: translation/ribosomal (334 genes, blue), amino acid metabolism (318 genes, green), general function (306 genes, yellow), transcription (263 genes, brown), inorganic ion transport (262 genes, purple), and cell wall/membrane biogenesis (260 genes, gray). The high density of AMR genes throughout the chromosome indicates extensive resistance potential against multiple antimicrobial classes

**Fig. 9:**
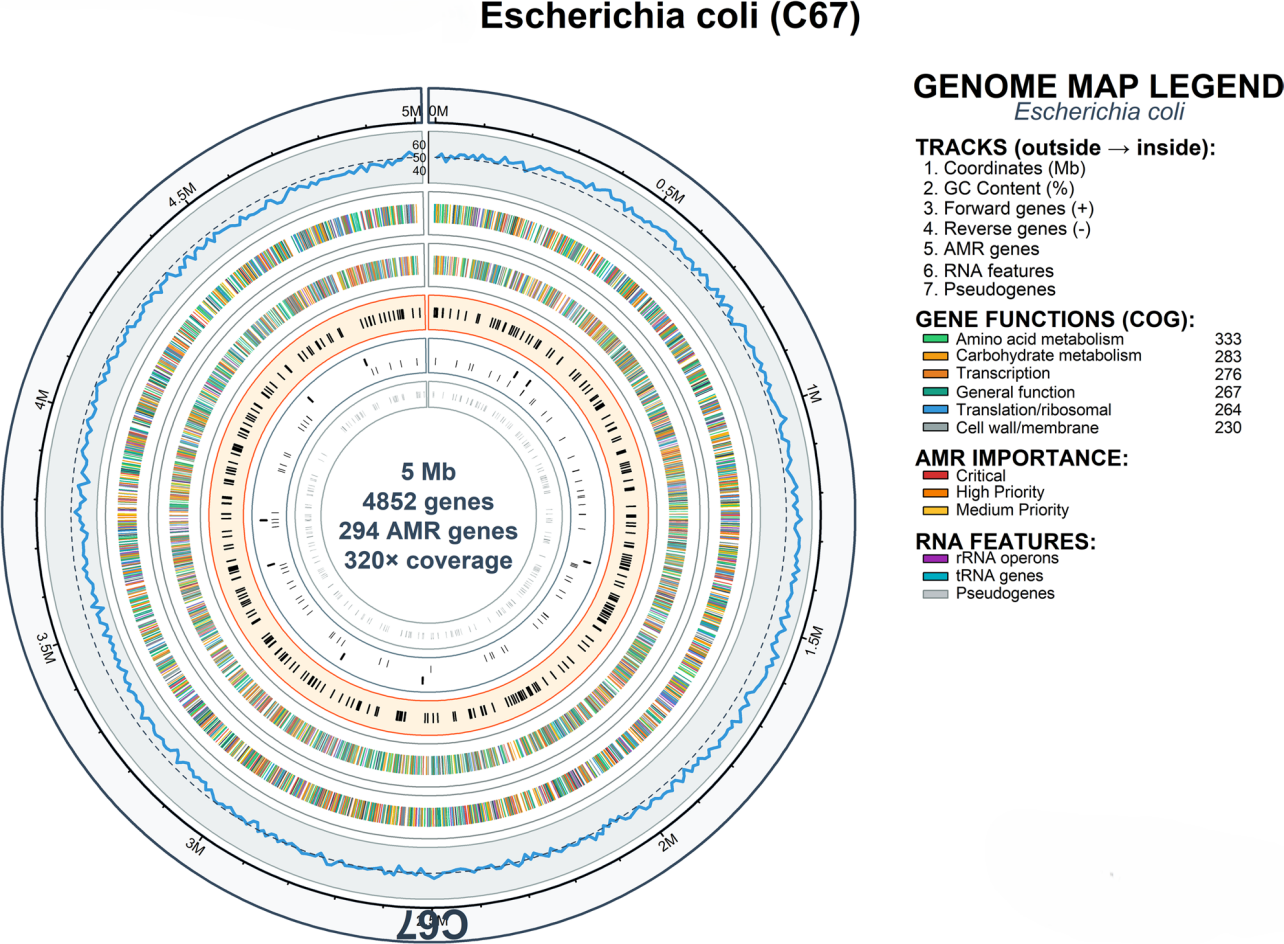
The circular representation illustrates the complete 5.0 Mb genome containing 4,852 genes with 294 antimicrobial resistance (AMR) genes distributed across the chromosome. Track organization follows the same pattern as Figure 8: (1) genome coordinates in megabases; (2) GC content percentage fluctuations shown as a blue line; (3) forward strand genes (+) with COG functional color coding; (4) reverse strand genes (-) with corresponding colors; (5) AMR genes categorized by clinical importance levels; (6) RNA features including rRNA operons, tRNA genes, and pseudogenes; and (7) innermost pseudogene track. Sequencing achieved 320× coverage depth. COG functional distribution shows: amino acid metabolism (333 genes, green), carbohydrate metabolism (283 genes, orange), transcription (276 genes, brown), general function (267 genes, yellow), translation/ribosomal (264 genes, blue), and cell wall/membrane (230 genes, gray). The extensive AMR gene complement (294 genes, 6.5% of protein-coding genes) demonstrates significant resistance potential, with genes distributed throughout the genome architecture rather than clustered in specific regions, suggesting multiple acquisition events and chromosomal integration of resistance determinants

The antimicrobial resistance genes identified in the two milk isolates represented ten distinct resistance classes. Isolate C65 harbored resistance genes across eight antimicrobial classes, whereas isolate C67 harbored resistance genes across 10 antimicrobial classes (Fig. 7 A). When considering individual resistance genes, C65 harbored 287 total AMR genes (6.7% of protein-coding genes) while C67 contained 294 total AMR genes (6.5% of protein-coding genes), with multiple genes often contributing to resistance within single antimicrobial classes. Comprehensive genomic analysis of C65 revealed a 4.75 Mb circular chromosome containing 4,442 genes, including 287 AMR genes distributed throughout the genome architecture (Fig. 8). Similarly, C67 demonstrated a complex genomic architecture with 4,852 total genes, including 294 AMR genes (6.5% of protein-coding genes), distributed across the Escherichia coli genome (Fig. 9). Both isolates were resistant to beta-lactam antibiotics, tetracyclines, aminoglycosides, sulfonamides, chloramphenicol, macrolides, glycopeptides, and quinolones. However, C67 uniquely possesses efflux pump genes and polymyxin resistance determinants.

Phylogenetic mapping revealed that AMR genes originated from four distinct bacterial orders: Enterobacterales, Bacillales, Enterococcales, and Pseudomonadales (Fig. 7B). Enterobacterales was the most prevalent source, contributing to beta-lactam, aminoglycoside, sulfonamide, and quinolone resistance. Quantitative similarity analysis using Jaccard coefficients revealed a similarity index of 0.800 between the isolates, with eight resistance classes (80%) shared and two unique to C67 (Fig. 7 C).

Phylogenetic analysis of resistance genes based on BLAST searches against the CARD database revealed that 61% showed highest similarity to genes from Proteobacteria-associated organisms and 39% to Firmicutes-associated organisms (Fig. 7D). The presence of resistance genes with phylogenetic origins from multiple bacterial orders (Enterobacterales, Bacillales, Pseudomonadales, and Enterococcales) and phyla in single isolates, combined with detection of mobile genetic elements (plasmids, integrons, and insertion sequences), suggests potential for horizontal gene transfer across taxonomic boundaries.

## Discussion

### Whole genome sequencing unveils hidden microbial complexity in bovine mastitis

This pilot study provides methodological validation for the integration of whole-genome sequencing into veterinary diagnostic workflows, demonstrating critical limitations in conventional identification approaches for environmental opportunistic pathogens. The misidentification of Stutzerimonas stutzeri as a gram-positive organism illustrates how environmental bacteria may exhibit phenotypic plasticity in host-associated environments, leading to systematic diagnostic errors when relying solely on morphological and biochemical characteristics. This highlights the limitations of phenotypic identification methods and emphasizes the importance of molecular confirmation, particularly for environmental bacteria that exhibit atypical morphological characteristics in clinical samples. This discovery exemplifies the critical limitations of culture-dependent identification approaches, which remain heavily biased toward detecting expected mastitis pathogens while systematically missing environmentally derived opportunistic organisms [1, 2].

Recent advances in culture-independent diagnostic approaches have revealed substantial microbial diversity in mastitis-affected quarters beyond that of traditionally recognized pathogens [28]. The identification of S. stutzeri in one mastitis case raises the hypothesis that environmental bacteria may be more prevalent in mastitis than currently recognized, as this organism has been largely overlooked in veterinary diagnostics that rely on conventional identification methods. The extensive antimicrobial resistance profile observed in this isolate (287 resistance genes comprising 6.7% of the genome) suggests that environmental opportunists may harbor substantial resistance potential, raising questions about their role as potential resistance reservoirs and facilitators of horizontal gene transfer within the mammary gland microbiome [5]. However, determination of the true prevalence and clinical significance of S. stutzeri in mastitis requires larger-scale surveillance studies employing whole-genome sequencing to identify isolates that conventional methods might miss.

### Diagnostic gaps and the need for genomic surveillance

The misidentification of isolate C65 in this case demonstrates that conventional microbiological methods optimized for common pathogens can fail to identify certain environmental bacteria with atypical phenotypic characteristics [24]. Whether such misidentifications represent systematic diagnostic gaps in mastitis surveillance or isolated cases cannot be determined from this single observation and requires prospective validation studies comparing conventional and genomic identification methods across larger, randomly selected sample sets from multiple diagnostic laboratories.

Whole metagenome sequencing studies have consistently revealed that 30% of the bacterial strains in clinical mastitis samples were previously unreported, highlighting the magnitude of unrecognized microbial diversity [13]. The implementation of culture-independent methods represents a critical evolution from a pathogen-focused to an ecosystem-level understanding of mastitis pathogenesis, enabling the detection of bacterial communities that traditional methods systematically miss.

### Antimicrobial resistance gene diversity and horizontal transfer potential

The extensive antimicrobial resistance gene repertoires identified (287–294 individual genes distributed across 8–10 resistance classes) demonstrate the complexity of resistance evolution in mixed microbial communities. The detection of resistance genes from four distinct bacterial orders (Enterobacterales, Bacillales, Enterococcales, and Pseudomonadales) within single isolates provides evidence of horizontal gene transfer potential through co-localization of resistance genes with mobile genetic elements. Both isolates demonstrated extensive genomic resistomes that far exceed phenotypically expressed resistance, indicating a substantial silent resistance potential [24, 25]. This phenomenon reflects the capacity for resistance gene activation under selective pressure, which poses significant challenges to antimicrobial stewardship in dairy farming.

Phylogenetic distribution analysis showing resistance genes with sequence similarity to genes from four distinct bacterial orders (Enterobacterales, Bacillales, Pseudomonadales, and Enterococcales) suggests potential for horizontal gene transfer across major taxonomic divisions. The observation that resistance genes in our isolates show phylogenetic affinity to both Proteobacteria (61%) and Firmicutes (39%), combined with the presence of mobile genetic elements, is consistent with horizontal gene transfer having occurred during the evolutionary history of these resistance determinants [27]. However, we acknowledge that our data based on phylogenetic distribution patterns do not provide direct evidence of active transfer events, recent acquisition, or detailed genomic context that would definitively demonstrate horizontal gene transfer mechanisms. Such determination would require comparative genomic analysis with closely related strains, synteny analysis, and characterization of flanking regions and genomic islands.

### Environmental reservoirs and one health implications

The identification of a multidrug-resistant environmental bacterium in one mastitis case raises questions about potential health implications if such organisms are more prevalent than currently recognized. Published literature indicates that dairy environments can serve as interface zones where bacterial communities from animals, humans, and the environment interact [11], potentially creating opportunities for resistance gene dissemination across these domains. Our observation of resistance genes to newer antimicrobials (ceftazidime, cefquinome, colistin) in this single isolate is consistent with broader surveillance data showing such resistance in bovine mastitis pathogens across multiple continents [22, 26]. However, our pilot study with two isolates cannot assess the clinical impact of environmental bacteria in mastitis, the transmission risk to human pathogens, or the contribution to the global resistance crisis without epidemiological follow-up and larger-scale surveillance.

Recent surveillance data from multiple continents have consistently demonstrated an increasing prevalence of methicillin-resistant staphylococci and extended-spectrum beta-lactamase producers in bovine mastitis [23, 31]. The mammary gland represents a unique ecological niche where environmental bacteria, commensals, and pathogens coexist under intermittent antimicrobial pressure, potentially accelerating the evolution and dissemination of resistance.

### Clinical and therapeutic implications

Genomic characterization of environmental mastitis pathogens reveals virulence factors and metabolic capabilities that provide crucial insights into pathogenesis mechanisms distinct from those of traditional mastitis-causing organisms [4]. Environmental bacteria that cause mastitis may respond differently to standard therapeutic protocols designed for conventional pathogens, potentially contributing to treatment failure and chronic infections. Comparative genomic studies across multiple continents have identified distinct regional variations in the strain distribution and resistance patterns, emphasizing the need for geographically tailored diagnostic and therapeutic approaches [20]. The extensive resistance profiles identified suggest that empirical antibiotic therapy may be inadequate for infections caused by environmentally derived pathogens.

### Study limitations and future directions

Several limitations of this study must be acknowledged when interpreting these results.First, the extremely limited sample size (*n* = 2 isolates representing 0.99% of positive samples) restricts the generalizability of the findings to a broader population of mastitis pathogens. This small sample size, while appropriate for a pilot proof-of-concept study, rules out robust statistical inference about the prevalence of misidentification or the frequency of extensive resistome profiles in the broader mastitis pathogen population. Second, the observed species identification discrepancy may represent an isolated case rather than a systematic diagnostic limitation of the method. Third, the cost constraints of whole-genome sequencing limited our ability to perform comprehensive genomic surveillance across multiple pathogen species and geographic locations. Fourth, while our data demonstrate phylogenetic diversity of resistance genes and the presence of mobile genetic elements, we did not perform detailed synteny analysis or comprehensive genomic context characterization to definitively demonstrate horizontal gene transfer events. Fifth, the non-random selection of isolates for whole-genome sequencing means that our findings cannot be extrapolated to estimate the true prevalence of misidentification or extensive resistomes in the broader mastitis pathogen population.

The search for therapeutic alternatives beyond traditional antibiotics has intensified with promising developments in herbal medicine, nanotechnology, polymers, and phototherapy, which have been shown to be effective against mastitis pathogens [19]. The integration of rapid molecular diagnostics with genomic surveillance represents a critical advancement in precision veterinary medicine, enabling species identification and resistance profiling within clinically relevant time frames.

Phylogenetic analyses based on whole-genome sequences provide unprecedented insights into the evolutionary relationships between mastitis pathogens, revealing patterns of virulence gene acquisition and resistance evolution [6]. Future surveillance programs should integrate metagenomic approaches with whole-genome sequencing of isolates to provide comprehensive insights into mammary gland microbiome dynamics and their roles in mastitis pathogenesis. Future studies should include larger, randomly selected sample sizes and comprehensive genotype-phenotype correlation analyses to validate these preliminary observations. As a pilot study, our findings should be interpreted as hypothesis-generating rather than hypothesis-testing research and the broader literature context is critical for appropriate interpretation and guides the design of future confirmatory studies.

## Conclusions

This pilot study provides preliminary evidence of the potential of whole genome sequencing to identify bacterial species that may be missed by conventional diagnostic methods for bovine mastitis. The identification of Stutzerimonas stutzeri as a misclassified environmental pathogen demonstrates the potential for systematic diagnostic errors when relying on phenotypic characteristics alone. The extensive antimicrobial resistance profiles identified, coupled with evidence of horizontal gene transfer potential between phylogenetically distant species, underscore the complexity of resistance evolution in dairy environments.

However, these findings are based on a limited sample size (*n* = 2 isolates from 202 positive samples) and require validation through large-scale studies before broader conclusions regarding diagnostic limitations can be drawn. Future research should include: (1) larger-scale WGS studies across diverse mastitis pathogen species to quantify misidentification rates, (2) prospective comparison of conventional versus genomic identification methods in routine diagnostic workflows, (3) cost-effectiveness analyses to assess the feasibility of implementing WGS in veterinary diagnostics, and (4) investigation of the clinical impact of species misidentification on treatment outcomes and antimicrobial stewardship. Despite these limitations, these findings support the potential utility of genomic surveillance programs in veterinary medicine to improve the accuracy of pathogen identification, guide evidence-based therapy, and monitor emerging resistance.

## Supplementary Information

Supplementary Material 1: Figure S1. Bioinformatics workflow for whole genome sequence analysis. Comprehensive pipeline showing quality control (Trimmomatic v0.39, FastQC, Kraken2), de novo assembly (SPAdes v3.15.4), quality assessment (QUAST, BUSCO, CheckM), species identification (FastANI, dDDH, 16S rRNA phylogenetics), genome annotation (NCBI PGAP), functional annotation (BLASTp, Blast2GO v5.2, KEGG KAAS, COG, Pfam), antimicrobial resistance analysis (CARD database), mobile genetic element detection (PlasmidFinder, IntegronFinder, ISfinder, ISEScan), and comparative genomic analysis. Color coding indicates analytical stages from input through final outputs. All software versions and parameters are shown.

Supplementary Material 2.

Supplementary Material 3.

## Data Availability

Raw sequencing data were deposited in the NCBI Sequence Read Archive under the BioProject PRJNA1048756 (accession numbers SRS25103899 and SRS25138938). The assembled genomes were submitted to GenBank under accession numbers GCA_050565245.1 and GCA_050565265.1. Complete antimicrobial resistance gene annotations with gene identities, CARD accession numbers, percentage identities, drug class assignments, and resistance mechanisms for all identified AMR genes are provided in Supplementary files S1 and S2. All bioinformatics workflows and custom analysis scripts were available upon request to ensure reproducibility.

## References

[1] Ahmadi A, Khezri A, Nørstebø H, Ahmad R. A culture-, amplification-independent, and rapid method for identification of pathogens and antibiotic resistance profile in bovine mastitis milk. Front Microbiol. 2022;13:1104701. 10.3389/fmicb.2022.1104701. 36687564 10.3389/fmicb.2022.1104701 PMC9852903

[2] Algharib SA, Dawood AS, Huang L, Guo A, Zhao G, Zhou K, et al. Basic concepts, recent advances, and future perspectives in the diagnosis of bovine mastitis. J Vet Sci. 2024;25:e18. 10.4142/jvs.23147. 38311330 10.4142/jvs.23147 PMC10839174

[3] Altschul SF, Madden TL, Schäffer AA, Zhang J, Zhang Z, Miller W, Lipman DJ. Gapped BLAST and PSI-BLAST: a new generation of protein database search programs. Nucleic Acids Res. 1997;25:3389–402. 10.1093/nar/25.17.3389. 9254694 10.1093/nar/25.17.3389 PMC146917

[4] Ashraf S, Naushad S, Si W, Bilal M, Ijaz M, Huang H, et al. Draft genome sequences and antimicrobial resistance genes of five Staphylococcus aureus strains isolated from bovine milk. Microbiol Resour Announc. 2022;11:e00756-22. 10.1128/mra.00756-22. 36190249 10.1128/mra.00756-22 PMC9584340

[5] Berendonk TU, Manaia CM, Merlin C, Fatta-Kassinos D, Cytryn E, Walsh F, Bürgmann H, Sørum H, Norström M, Pons M-N, Kreuzinger N, Huovinen P, Stefani S, Schwartz T, Kisand V, Baquero F, Martinez JL. Tackling antibiotic resistance: the environmental framework. Nat Rev Microbiol. 2015;13:310–7. 10.1038/nrmicro3439. 25817583 10.1038/nrmicro3439

[6] Crippa BL, Rodrigues MX, Tomazi T, Yang Y, de Oliveira Rocha L, Bicalho RC, et al. Virulence factors, antimicrobial resistance and phylogeny of bovine mastitis-associated Streptococcus dysgalactiae. J Dairy Res. 2023;90:152–7. 10.1017/S0022029923000195. 37042313 10.1017/S0022029923000195

[7] Ellington MJ, Ekelund O, Aarestrup FM, Canton R, Doumith M, Giske C, et al. The role of whole genome sequencing in antimicrobial susceptibility testing of bacteria: report from the EUCAST subcommittee. Clin Microbiol Infect. 2017;23:2–22. 10.1016/j.cmi.2016.11.012. 27890457 10.1016/j.cmi.2016.11.012

[8] Falentin H, Rault L, Nicolas A, Bouchard DS, Lassalas J, Lamberton P, et al. Bovine teat microbiome analysis revealed reduced alpha diversity and significant changes in taxonomic profiles in quarters with a history of mastitis. Front Microbiol. 2016;7:480. 10.3389/fmicb.2016.00480. 27242672 10.3389/fmicb.2016.00480 PMC4876361

[9] Forsberg KJ, Reyes A, Wang B, Selleck EM, Sommer MOA, Dantas G. The shared antibiotic resistome of soil bacteria and human pathogens. Science. 2012;337:1107–11. 10.1126/science.1220761. 22936781 10.1126/science.1220761 PMC4070369

[10] G O, Ml B, Re EM, Vs RCF, Ag M, Yh TCS, Rc S, B. Microbiota of cow’s milk; distinguishing healthy, sub-clinically and clinically diseased quarters. PLoS ONE. 2014;9. 10.1371/journal.pone.0085904. 10.1371/journal.pone.0085904 PMC3896433 24465777

[11] Guardabassi L, Butaye P, Dockrell DH, Fitzgerald JR, Kuijper EJ, ESCMID Study Group for Veterinary Microbiology (ESGVM). One health: a multifaceted concept combining diverse approaches to prevent and control antimicrobial resistance. Clin Microbiol Infect. 2020;26:1604–5. 10.1016/j.cmi.2020.07.012. 32702500 10.1016/j.cmi.2020.07.012

[12] Hendriksen RS, Bortolaia V, Tate H, Tyson GH, Aarestrup FM, McDermott PF. Using genomics to track global antimicrobial resistance. Front Public Health. 2019;7. 10.3389/fpubh.2019.00242. 10.3389/fpubh.2019.00242 PMC6737581 31552211

[13] Hoque MN, Istiaq A, Clement RA, Gibson KM, Saha O, Islam OK, et al. Insights into the resistome of bovine clinical mastitis microbiome, a key factor in disease complication. Front Microbiol. 2020;11:860. 10.3389/fmicb.2020.00860. 32582039 10.3389/fmicb.2020.00860 PMC7283587

[14] Jain C, Rodriguez-R LM, Phillippy AM, Konstantinidis KT, Aluru S. High throughput ANI analysis of 90K prokaryotic genomes reveals clear species boundaries. Nat Commun. 2018;9:5114. 10.1038/s41467-018-07641-9. 30504855 10.1038/s41467-018-07641-9 PMC6269478

[15] Jia B, Raphenya AR, Alcock B, Waglechner N, Guo P, Tsang KK, Lago BA, Dave BM, Pereira S, Sharma AN, Doshi S, Courtot M, Lo R, Williams LE, Frye JG, Elsayegh T, Sardar D, Westman EL, Pawlowski AC, Johnson TA, Brinkman FSL, Wright GD, McArthur AG. CARD 2017: expansion and model-centric curation of the comprehensive antibiotic resistance database. Nucleic Acids Res. 2017;45:D566–73. 10.1093/nar/gkw1004. 27789705 10.1093/nar/gkw1004 PMC5210516

[16] Jl M, Tm C, F, B. What is a resistance gene? Ranking risk in resistomes. Nat Rev Microbiol. 2015;13. 10.1038/nrmicro3399. 10.1038/nrmicro3399 25534811

[17] Köser CU, Ellington MJ, Cartwright EJP, Gillespie SH, Brown NM, Farrington M, et al. Routine use of microbial whole genome sequencing in diagnostic and public health microbiology. PLoS Pathog. 2012;8:e1002824. 10.1371/journal.ppat.1002824. 22876174 10.1371/journal.ppat.1002824 PMC3410874

[18] Kuehn JS, Gorden PJ, Munro D, Rong R, Dong Q, Plummer PJ, et al. Bacterial community profiling of milk samples as a means to understand culture-negative bovine clinical mastitis. PLoS One. 2013;8:e61959. 10.1371/journal.pone.0061959. 23634219 10.1371/journal.pone.0061959 PMC3636265

[19] Kuralkar P, Kuralkar SV. Role of herbal products in animal production - an updated review. J Ethnopharmacol. 2021;278:114246. 10.1016/j.jep.2021.114246. 34052352 10.1016/j.jep.2021.114246

[20] Lippolis JD, Holman DB, Brunelle BW, Thacker TC, Bearson BL, Reinhardt TA, et al. Genomic and transcriptomic analysis of *Escherichia coli* strains associated with persistent and transient bovine mastitis and the role of colanic acid. Infect Immun. 2017;86:e00566-17. 10.1128/IAI.00566-17. 29061709 10.1128/IAI.00566-17 PMC5736815

[21] Meier-Kolthoff JP, Carbasse JS, Peinado-Olarte RL, Göker M. TYGS and LPSN: a database tandem for fast and reliable genome-based classification and nomenclature of prokaryotes. Nucleic Acids Res. 2022;50:D801–7. 10.1093/nar/gkab902. 34634793 10.1093/nar/gkab902 PMC8728197

[22] Molineri AI, Camussone C, Zbrun MV, Suárez Archilla G, Cristiani M, Neder V, et al. Antimicrobial resistance of Staphylococcus aureus isolated from bovine mastitis: systematic review and meta-analysis. Prev Vet Med. 2021;188:105261. 10.1016/j.prevetmed.2021.105261. 33508662 10.1016/j.prevetmed.2021.105261

[23] Mostafa Abdalhamed A, Zeedan GSG, Ahmed Arafa A, Shafeek Ibrahim E, Sedky D, Abdel Nabey Hafez A. Detection of methicillin-resistant *Staphylococcus aureus* in clinical and subclinical mastitis in ruminants and studying the effect of novel green synthetized nanoparticles as one of the alternative treatments. Vet Med Int. 2022;2022:6309984. 10.1155/2022/6309984. 36457891 10.1155/2022/6309984 PMC9708356

[24] Naranjo-Lucena A, Slowey R. Invited review: antimicrobial resistance in bovine mastitis pathogens: a review of genetic determinants and prevalence of resistance in European countries. J Dairy Sci. 2023;106:1–23. 10.3168/jds.2022-22267. 36333144 10.3168/jds.2022-22267

[25] Naushad S, Nobrega DB, Naqvi SA, Barkema HW, De Buck J. Genomic analysis of bovine *Staphylococcus aureus* isolates from milk to elucidate diversity and determine the distributions of antimicrobial and virulence genes and their association with mastitis. mSystems. 2020;5:e00063-20. 10.1128/mSystems.00063-20. 32636332 10.1128/mSystems.00063-20 PMC7343304

[26] Oliver SP, Murinda SE. Antimicrobial resistance of mastitis pathogens. Vet Clin North Am Food Anim Pract. 2012;28:165–85. 10.1016/j.cvfa.2012.03.005. 22664201 10.1016/j.cvfa.2012.03.005

[27] Ronco T, Klaas IC, Stegger M, Svennesen L, Astrup LB, Farre M, Pedersen K. Genomic investigation of Staphylococcus aureus isolates from bulk tank milk and dairy cows with clinical mastitis. Vet Microbiol. 2018;215:35–42. 10.1016/j.vetmic.2018.01.003. 29426404 10.1016/j.vetmic.2018.01.003

[28] Rötzer V, Wenderlein J, Wiesinger A, Versen F, Rauch E, Straubinger RK, et al. Bovine udder health: from standard diagnostic methods to new approaches—a practical investigation of various udder health parameters in combination with 16S rRNA sequencing. Microorganisms. 2023;11:1311. 10.3390/microorganisms11051311. 37317285 10.3390/microorganisms11051311 PMC10221688

[29] Ruegg PL. A 100-year review: mastitis detection, management, and prevention. J Dairy Sci. 2017;100:10381–97. 10.3168/jds.2017-13023. 10.3168/jds.2017-13023 29153171

[30] Taponen S, Pyörälä S. Coagulase-negative staphylococci as cause of bovine mastitis- not so different from Staphylococcus aureus? Vet Microbiol. 2009;134:29–36. 10.1016/j.vetmic.2008.09.011. 10.1016/j.vetmic.2008.09.011 18977615

[31] Yang F, Shi W, Meng N, Zhao Y, Ding X, Li Q. Antimicrobial resistance and virulence profiles of staphylococci isolated from clinical bovine mastitis. Front Microbiol. 2023. 10.3389/fmicb.2023.1190790. 10.3389/fmicb.2023.1190790 PMC10344457 37455736

