# Supplementary figures and images for: Whole genome sequencing reveals environmental pathogen misidentification and potential for cross-phylum antimicrobial resistance gene transfer in bovine mastitis: a pilot genomic study
Source: BMC Vet Res. 2026 Jan 14;22:134. doi: 10.1186/s12917-025-05280-z (PMC12933902; doi:10.1186/s12917-025-05280-z)

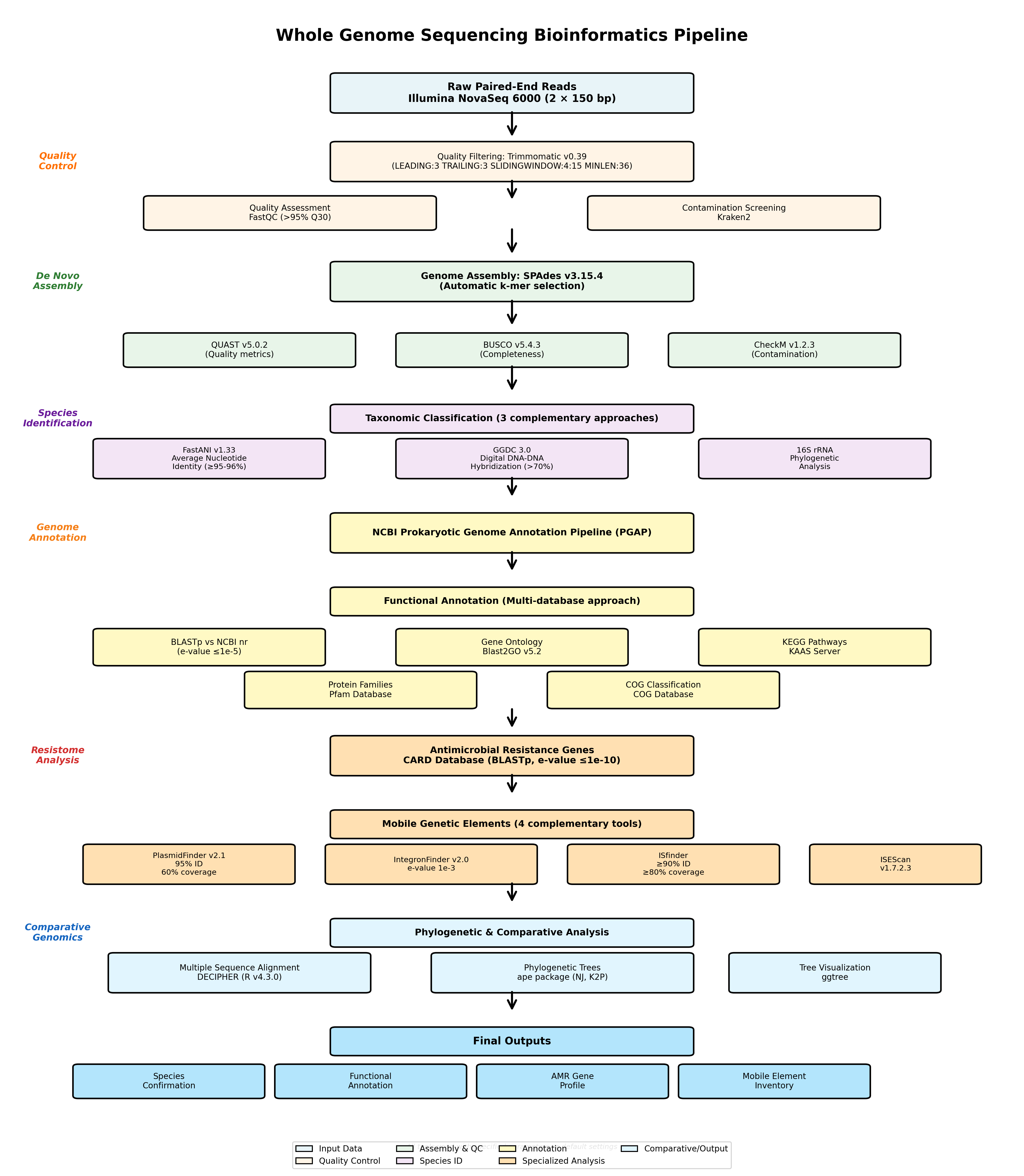

Supplement: Supplementary file 1 — Supplementary Material 1: Figure S1. Bioinformatics workflow for whole genome sequence analysis. Comprehensive pipeline showing quality control (Trimmomatic v0.39, FastQC, Kraken2), de novo assembly (SPAdes v3.15.4), quality assessment (QUAST, BUSCO, CheckM), species identification (FastANI, dDDH, 16S rRNA phylogenetics), genome annotation (NCBI PGAP), functional annotation (BLASTp, Blast2GO v5.2, KEGG KAAS, COG, Pfam), antimicrobial resistance analysis (CARD database), mobile genetic element detection (PlasmidFinder, IntegronFinder, ISfinder, ISEScan), and comparative genomic analysis. Color coding indicates analytical stages from input through final outputs. All software versions and parameters are shown. [file 12917_2025_5280_MOESM1_ESM.png]
